# Is Silicon a Panacea for Alleviating Drought and Salt Stress in Crops?

**DOI:** 10.3389/fpls.2020.01221

**Published:** 2020-08-18

**Authors:** Sarah J. Thorne, Susan E. Hartley, Frans J. M. Maathuis

**Affiliations:** ^1^Department of Biology, University of York, York, United Kingdom; ^2^Department of Biology, University of Sheffield, Sheffield, United Kingdom

**Keywords:** agronomy, drought, osmotic stress, oxidative stress, photosynthesis, review, salinity, silicon fertilization

## Abstract

Salinity affects around 20% of all arable land while an even larger area suffers from recurrent drought. Together these stresses suppress global crop production by as much as 50% and their impacts are predicted to be exacerbated by climate change. Infrastructure and management practices can mitigate these detrimental impacts, but are costly. Crop breeding for improved tolerance has had some success but is progressing slowly and is not keeping pace with climate change. In contrast, Silicon (Si) is known to improve plant tolerance to a range of stresses and could provide a sustainable, rapid and cost-effective mitigation method. The exact mechanisms are still under debate but it appears Si can relieve salt stress *via* accumulation in the root apoplast where it reduces “bypass flow of ions to the shoot. Si-dependent drought relief has been linked to lowered root hydraulic conductance and reduction of water loss through transpiration. However, many alternative mechanisms may play a role such as altered gene expression and increased accumulation of compatible solutes. Oxidative damage that occurs under stress conditions can be reduced by Si through increased antioxidative enzymes while Si-improved photosynthesis has also been reported. Si fertilizer can be produced relatively cheaply and to assess its economic viability to improve crop stress tolerance we present a cost-benefit analysis. It suggests that Si fertilization may be beneficial in many agronomic settings but may be beyond the means of smallholder farmers in developing countries. Si application may also have disadvantages, such as increased soil pH, less efficient conversion of crops into biofuel and reduced digestibility of animal fodder. These issues may hamper uptake of Si fertilization as a routine agronomic practice. Here, we critically evaluate recent literature, quantifying the most significant physiological changes associated with Si in plants under drought and salinity stress. Analyses show that metrics associated with photosynthesis, water balance and oxidative stress all improve when Si is present during plant exposure to salinity and drought. We further conclude that most of these changes can be explained by apoplastic roles of Si while there is as yet little evidence to support biochemical roles of this element.

## Introduction

Drought and salinity stress lead to water deficits, ionic toxicity, nutrient imbalances, and the occurrence of oxidative stress. General responses include reduced growth, which is reflected in downregulation of genes that encode proteins involved in cell wall expansion, protein synthesis, and DNA synthesis (Skirycz and Inzé, 2010). Photosynthesis is also inhibited with photosynthetic genes that encode specific components of photosystem I and II concomitantly downregulated (Chaves et al., 2009). Dehydration and a perturbed ion balance require transcriptional regulation of aquaporins and ion transport functions to accommodate changes in water status and ionic content (Maathuis et al., 2003; Luu and Maurel, 2005; Alexandersson et al., 2010). Osmotic imbalance leads to the uptake of inorganics and the biosynthesis of compatible solutes (Bohnert and Shen, 1998). Protein ubiquitination becomes more prevalent, symptomatic of generally increased protein turnover and a reshaping of the proteome. The generation of reactive oxygen species (ROS) during drought and salinity stress requires upregulation of detoxification systems such as super oxide dismutase (SOD) and catalase enzymes and biosynthesis of ROS scavengers (You and Chan, 2015). Bioenergetics are modulated to serve changing energy demands for instance for the extrusion and compartmentation of ions.

Around 20% of all arable land is negatively affected by salt stress (FAO and ITPS, 2015), while drought stress has been estimated to reduce average cereal yields by 10% (Lesk et al., 2016). Moreover, drought and salinity stress are expected to become more common due to climate change (IPCC, 2014). New strategies to mitigate against the detrimental impacts of drought and salinity stress are urgently needed to ensure future food security. To date, crop breeding and genetic modification (GM) have had limited success at developing new high-yielding cultivars with high stress tolerance (Sallam et al., 2019). Crop irrigation systems are expensive and can negatively impact on water availability in other areas (Martínez-Alvarez et al., 2016). Alternatively, Si fertilization could provide a relatively cheap method of improving crop stress tolerance. In this review, we briefly describe the response of plants to drought and salinity stress. We then discuss the potential of using Si fertilization to improve plant stress tolerance. We focus on the most significant physiological changes associated with Si in plants under drought and salinity stress, aiming to quantify the average effect of Si measured in recent studies. While the exact mechanism underpinning the benefits of Si remains debated, we explore how the majority of Si effects can be attributed to the deposition of Si in the apoplast, with a current lack of evidence supporting a biochemical role for Si. Finally, we assess the economic feasibility of using Si fertilizer.

## Drought

Drought is generally defined as a lack of water that leads to stress symptoms in plants. In an agronomic context it could be defined as any water deficit that reduces crop production to below its optimum yield. Among key crops, it is estimated that around 75% of global harvested area experiences yield losses due to drought stress (Kim et al., 2019). Drought happens in almost any habitat, albeit with varying frequency and severity, and is responsible for global yield reductions in wheat and maize of around 20 and 40% respectively (Daryanto et al., 2016). This ubiquitous occurrence has contributed to a wide panoply of plant adaptations and responses that take place across timescales from seconds to days, or even weeks. The most prominent of these are briefly discussed below in chronological order.

### Perception and Early Responses

Although the exact mechanism is still debated, there are now several credible molecular candidates that act as primary sensors of drought and osmotic stress, such as the histidine kinase HK1 and the Ca^2+^ permeable channel OSCA1 (Yuan et al., 2014). In case of the former, the HK1 receptor kinase has a cell wall binding motif whereas other domains reside in the plasma membrane. Relative repositioning of these components by changes in cellular volume are therefore reported by the kinase and subsequently cause its autophosphorylation and initiation of downstream phosphorelays. Osmotic stress has long been known to generate Ca^2+^ signals within seconds and the mechanosensitive OSCA1 ion channel transduces the drought-induced membrane deformations into a Ca^2+^ influx. Perception is likely followed within minutes by transcriptional and post-translational modulation of gene and protein expression.

### Gene Regulation

Early signals are transmitted by a variety of secondary messengers such as Ca^2+^, ROS, hormones such as ABA (abscisic acid), and phospholipids. They involve kinase relays and ultimately target a large number of drought-responsive genes (Johnson et al., 2014). The latter typically encode functional proteins that include chaperones to protect soluble proteins and maintain membrane integrity, late embryogenesis abundant (LEA) proteins, antioxidants, osmotins, and proteins associated with the uptake and distribution of water, inorganic osmolytes, and organic osmolytes, such as aquaporins, sugar transporters and ion transporters (Hu and Xiong, 2014). Transcriptional adjustment heavily relies on the activity of hormones such as ABA, cytokinin (CK), gibberellic acid (GA), auxin, and ethylene (Tiwari et al., 2017). Drought induces biosynthesis of the major stress hormone ABA in the root from where it is translocated to the shoot and instigates stomatal closure. CK prevents leaf senescence while auxins are generally negative regulators of drought responses and promote expression of LEAs. The role of ABA has been studied in detail and this hormone typically alters transcription of relevant proteins *via* transcription factors that bind to cis-regulatory elements known as ABA-responsive elements (ABREs) (Liu et al., 2018). Transcription factors of multiple families contribute to this but many belong to the subfamily of bZIPs such as bZIP1 from sweet potato which was shown to be involved in both drought and salinity responses (Kang et al., 2019). Regulation of ABA-independent genes often occurs through the drought responsible element (DRE) and C-repeat (CRT) cis-acting elements which provide docking targets for DRE-binding protein or C-repeat-binding factor (CBF) transcription factors (Liu et al., 2018).

### Whole Plant Level Responses

Water deficits reduce plant growth but less so in the root than the shoot. A major role of ABA is in restoration of water balance in the plant. This is primarily achieved by reducing stomatal conductance and hence lowering transpirational flux to preserve water (Mittler and Blumwald, 2015). At the same time, ABA and other mechanisms can affect water conductance in root tissues for example by altering aquaporin expression to modulate water uptake. Alternatively or in parallel, suberisation in the exo- and endodermes can be accelerated to prevent the desiccation of inner tissue layers (Henry et al., 2012) though it would reduce the overall capacity of roots to take up water. Lowering the cellular water potential is another major adjustment which can be achieved by augmented uptake of inorganics, typically K^+^, and the biosynthesis of compatible solutes. Depending on species, the latter consist of amino acids like proline and beta-glycine or sugars. At the whole plant level, water deficit tends to create shifts in growth patterns favoring higher root:shoot ratios, a phenomenon that has been observed in many crops (e.g. see Kulkarni et al., 2017 for wheat). Greater root growth is often paralleled by changes in root architecture that promote proliferation of fine laterals allowing the exploration of deeper soil strata. Morphological changes in the shoot to reduce water loss can occur such as reduced stomatal density and the deposition of waxy surfaces in the cuticle (Bi et al., 2017). Water deficits reduce root growth and cause a pronounced suberisation of the apoplast and, perhaps, also affect the cellular passage of water *via* aquaporins. This influences the water balance by reducing the capacity of roots to take up water.

## Salinity

As is the case for drought, salinity greatly depresses agricultural yields with estimates of around 55% in maize and 28% in wheat (Satir and Berberoglu, 2016). A large component of salt stress consists of osmotic effects which create water deficits and a general disruption of water balance. Thus, almost all of the above mentioned responses are also pertinent in saline environments. However, salt stress is compounded by ionic toxicity which can affect the cellular biochemical machinery and generate nutrient stress. The latter are caused by the disproportionate presence of Na^+^, and to a lesser extent Cl^-^, in both cellular and extracellular compartments. In the cytosol, Na^+^ can substitute K^+^ which is essential for the activation of many enzymes. Uptake of essential nutrients like K^+^ and NO_3_^-^ can be disturbed by competitive inhibition of transport activity by the chemically similar Na^+^ and Cl^-^ ions. In the apoplast, ionic interactions of Ca^2+^ with cell wall pectins or membrane phospholipids can be disrupted by apoplastic Na^+^.

### Perception and Early Responses

Recent work identified a specific class of membrane sphingolipids as potential salt sensors. The lipids can bind monovalent cations which reduces the surface potential which in turn activates voltage dependent Ca^2+^ permeable ion channels. This mechanism could fill in the blanks that existed regarding upstream components of the salt dependent SOS-Ca^2+^ signaling pathway which is instrumental in regulating Na^+^ transport (Zhu, 2002). A second, cGMP-based pathway has also been reported which impacts on transport functions and gene transcription (Isner and Maathuis, 2018).

### Ion Transport

To cope with ionic toxicity, plants can reduce net influx of ions by reducing unidirectional ion uptake or increased ion efflux. Detoxification also involves the sequestration of Na^+^ and Cl^-^ in the vacuole which has as added benefit a lowering of the water potential. The membrane transporters involved in these processes have been identified and characterized and for many detailed information is available regarding their regulation. Unidirectional Na^+^ uptake from the soil is likely mediated by multiple systems including non-selective ion channels and HKT type carriers (Isayenkov and Maathuis, 2019). Na^+^ extrusion from the cytoplasm is likely carried out by Na^+^:H^+^ antiporters such as SOS1 while vacuolar sequestration is carried out by a similar antiporter from the NHX subfamily. HKTs can also retrieve Na^+^ from the xylem and as such limit salt translocation to the shoot.

SOS1 is a main target of the SOS pathway; SOS1 activity is elevated after phosphorylation by the kinase SOS2 while SOS2 itself associates with SOS3 a Ca^2+^-binding protein (Liu and Zhu, 1997). SOS2 may also affect the activity of other transporters such as HKTs involved in Na^+^ uptake and NHXs which are responsible for vacuolar Na^+^ loading. In the case of cGMP, it lowers Na^+^ uptake by reducing the activity of non-selective ion channels, a major uptake pathway. In addition to the roles of ABA described above, this hormone is also involved in regulation of ion transport as exemplified by regulation of NHX1 transcription and activity. ABA likely alters *NHX1* transcription *via* the phosphatase ABI1 and MYC/MYB transcription factors (Chinnusamy et al., 2004).

### Whole Plant Responses

In response to salinity, plants show different traits; in many glycophytes there is a strong negative correlation between plant growth and shoot Na^+^ concentrations. Such plants typically show ‘excluder’ behavior and limit Na^+^ levels in the shoot either through reduced xylem loading or *via* recirculation of Na^+^ from shoot to root by the phloem. Xylem loading is a function of ions that reach the stele and their subsequent trans-membrane flux mediated by proteins located in the xylem parenchyma. Most ions reach the stele *via* the root symplast due to the barrier function of the endodermal Casparian strip. However, in younger, less developed root regions the so-called “bypass flow” allows movement of solutes through the cell walls to the xylem without crossing a membrane. During saline conditions, the suberisation and lignification around endodermal cells can help prevent apoplastic uptake of toxic ions; this has indeed been observed in several species including beans, maize and cotton (Chen T. et al., 2011). Bypass flux may differ between seminal and lateral roots; For example, rice lateral roots do not have an exodermis and consequently may be responsible for the majority of apoplastic entry (Faiyue et al., 2012). As is the case for drought, salinity rapidly reduces transpiration by reducing both stomatal and root conductance. The latter will be brought about by a mixture of physical adaptation (e.g. suberisation of the endodermis) and regulation of aquaporin activity.

## Strategies for Mitigating Drought and Salt Stress

Mitigation of the negative impact of drought and salt stress requires multipronged tactics ranging from approaches at the plant molecular to the regional, or even national, level.

### Breeding

Farmers have carried out selection and breeding for tolerance across millennia. More recently, this process has been accelerated by focussing on specific genes and/or markers which have often been identified *via* forward or reverse screens. Modern breeding also benefits from exploiting genetic diversity, for example by using QTL (quantitative trait loci) and GWAS (genome wide association studies). Breeding and selection for adaptations to drought and salinity is difficult due to the multigenic basis of these stresses and the complex genotype by environment interactions. In addition, plants with high drought tolerance (xerophytes) are slow growing. This is less the case for halophytes but does imply that, at least for drought, tolerance and high growth rates are likely to be incompatible.

### Engineering and GM

There are many examples where the activity of genes that are relevant to drought and salinity has been manipulated to improve tolerance (reviewed in Yang et al., 2010; Roy et al., 2014). In several cases these approaches have been highly successful, even in field settings, while in other cases promising results were only obtained in lab surroundings (Flowers and Colmer, 2008).

Engineering and gene-editing approaches are potentially much faster than breeding but do require functional knowledge about the genes that contribute to tolerance, and gaining such knowledge can be laborious and time consuming. Other disadvantages are the continuing consumer protest to GM crops and the multigenic nature of drought and salt stress which implies that many ‘positive’ genes need to be combined (‘stacked’) to significantly enhance resilience to drought and salinity.

### Infrastructure and Agronomic Management

In the case of either stress, access to fresh water is essential and in general, water availability remains the most widespread limitation to crop yields. At a regional or national scale, irrigation systems can require considerable investment in the form of reservoirs and canal networks for water storage and distribution. Irrigation often relies on damming and rerouting of major rivers that serve multiple communities, and thus increases the risk of conflict over water resources. At a local scale farmers must rely on wells and surface waters in the immediate area to tackle drought spells but these supplies tend to be less reliable and provide limited capacity, especially during periods of drought.

Seawater ingression, one of the main causes of coastal salinization, can be prevented by building dykes while treatment with gypsum can help revitalize saline soils. In the case of moderate salinization, irrigation with high quality fresh water can to some extent leach out excess salts. However, prolonged excessive irrigation, especially in combination with poor drainage, is itself a principal cause of inland salinization (Thiruchelvam and Pathmarajah, 1999). Precision (drip) irrigation can drastically reduce fresh water consumption helping to preserve this increasingly scarce resource.

## Silicon—A Sustainable Alternative?

A further alternative to the above strategies is Silicon (Si) fertilization, which has been shown to improve plant tolerance to an array of biotic and abiotic stresses, including drought and salinity (reviewed in Debona et al., 2017; Luyckx et al., 2017; Frew et al., 2018). Si fertilization could provide farmers with a quick and cheap method of improving crop yield, in contrast to the above methods which are often slow to take effect and too expensive to be implemented by small-holder farmers. Furthermore, the benefits of Si fertilization have been found in a range of species including the major crops: rice, wheat, maize, and barley.

### The Mechanism of Si Accumulation

Si is the second most abundant element in the earth’s crust after oxygen. Si is present in the soil in a variety of forms, with SiO_2_ being the most prevalent (Sommer et al., 2006). However, plants only absorb Si from the soil in the form of silicic acid (Si(OH)_4_), which is often limiting in the soil (Côté-Beaulieu et al., 2009). Typically, soils contain 100–500 µmol L^-1^ silicic acid, although the exact availability varies depending on soil type, temperature, and pH (Sommer et al., 2006).

Plants vary significantly in their ability to accumulate Si from the soil, with rice accumulating up to 10% Si by dry weight (Epstein, 1994). A meta-analysis of over 700 plant species found that, in general, liverworts and horsetails accumulate more Si than angiosperms and gymnosperms, although there are exceptions to this (Hodson et al., 2005). Several important crop species, including wheat, barley, maize, and rice, are Si accumulators (Guntzer et al., 2012). Nevertheless, some dicots accumulate significant levels of Si, and there is significant variation in Si accumulation even within plant families (Katz, 2014).

**Figure 1:**
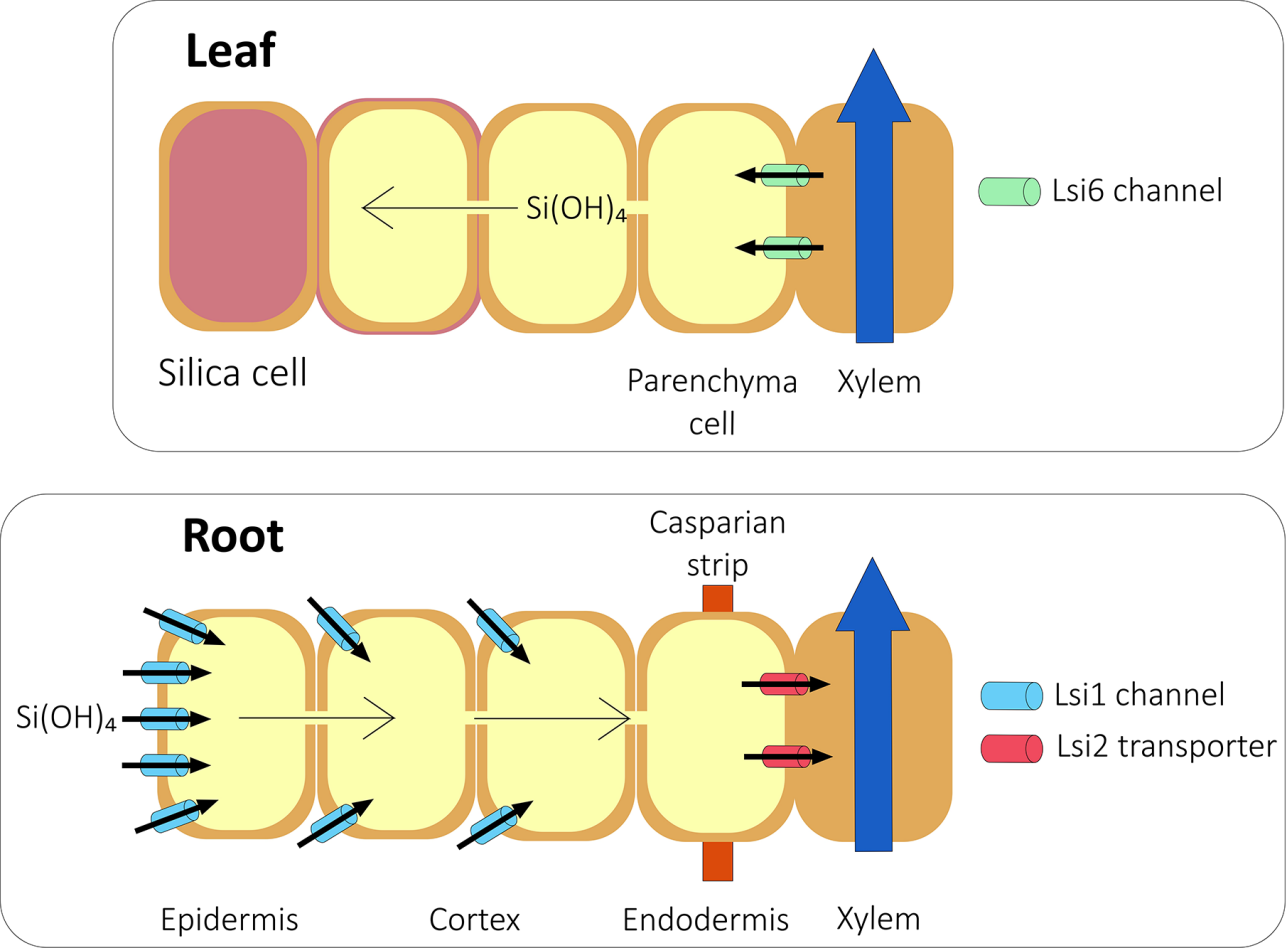
Si transport in a typical grass species. Silicic acid from the soil is transported into the root symplast by the action of aquaporins such as Lsi1 channels. The silicic acid then diffuses across the root into the endodermis. At the endodermis, Lsi2 transports silicic acid into the stelar apoplast from where it diffuses into the xylem and is transported to the shoot in the transpiration stream. In rice, the presence of aerenchyma means that Lsi2 is localized at both the exodermis and endodermis. In the shoot, silicic acid is unloaded from the xylem by further aquaporins such as Lsi6 and deposited in the cell walls and in specific silica cells. *Based on* Ma and Yamaji, 2015.

Although some Si is accumulated passively (Mitani and Ma, 2005; Exley, 2015; Kumar et al., 2017), there is abundant evidence that in Si accumulators, most Si is actively accumulated (Jarvis, 1987; Casey et al., 2003; Rains et al., 2006; Sun et al., 2016; McLarnon et al., 2017). In Si accumulators, two Si transporters, Lsi1 and Lsi2 (**Figure 1**), are needed to transport silicic acid from the soil through the root (Ma et al., 2006; Ma et al., 2007). A homolog of Lsi1, Lsi6, is needed to unload silicic acid from the xylem and into the shoot (**Figure 1**; Yamaji et al., 2008; Mitani et al., 2009; Yamaji et al., 2012).

Lsi1 has been characterized in several species including rice (Ma et al., 2006), wheat (Montpetit et al., 2012), barley (Chiba et al., 2009), maize (Mitani et al., 2009), pumpkin (Mitani et al., 2011), soybean (Deshmukh et al., 2013), and cucumber (Wang H. S. et al., 2015). Lsi1 is a NIP III aquaporin, a family characterized by a unique GSGR ar/R selectivity filter (Mitani et al., 2008). Lsi1 has six transmembrane domains, two NPA motifs, and is localized to the plasma-membrane (Ma et al., 2006). Lsi1 is highly specific for Si uptake, with the substrate specificity largely the result of the ar/R selectivity filter within the Lsi1 protein (Mitani et al., 2008) and a precise 108 amino acid spacing between NPA domains (Deshmukh et al., 2015).

Lsi2 is a member of an anion transporter family. It has eleven predicted transmembrane domains and is localized to the plasma membrane (Ma et al., 2007). Lsi2 functions as an efflux transporter, most likely in the form of an antiporter that exchanges Si with protons (Ma et al., 2007). The expression pattern of *OsLsi2* is similar to that of *OsLsi1* (Yamaji and Ma, 2011) and homologs of *OsLsi2* have been identified in maize and barley (Mitani et al., 2009), as well as in pumpkin (Mitani-Ueno et al., 2011) and cucumber (Sun et al., 2018). The genes encoding Si efflux transporters in horsetail have also been identified, although they share only low sequence similarity with the *Lsi2* genes of higher plants (Vivancos et al., 2016).

Ultimately, there are several destinations for Si once it has entered the plant symplast and this has been mostly studied in grasses. In roots, Si is mostly found in endo- and exo-dermal tissues where it could be integrated into the cell wall by cross linking with other wall components such as hemicelluloses, pectins and phenolics (Sakai and Thom, 1979; Fleck et al., 2015; He et al., 2015). In the shoot, high levels of silicic acid result in its autopolymerisation into silica (Yoshida et al., 1962a). Deposited silica can be found in the form of phytoliths which occur in a multitude of shoot tissues (reviewed in Shakoor et al., 2014). Alternatively, silica accumulates in or beneath the cuticle layer of the cell wall in epidermal cell layers and tissues that surround the vasculature (Yoshida et al., 1962b; Sakai and Thom, 1979; Peleg et al., 2010; Kumar et al., 2017).

## Mechanisms of Si-Induced Drought and Salt Tolerance

To maximize the potential benefits of Si fertilization, and assess the economic feasibility of large scale Si application, it is imperative that we understand the underlying mechanisms of how this element generates its effects. However, the literature reports a bewildering panoply of processes that appear to be affected by Si and there remains considerable debate regarding their relative importance for the mitigation of drought and salinity stress. We have therefore carried out a comprehensive literature search to produce a quantitative assessment of the different physiological and growth parameters altered by Si addition in plants under drought and salinity stress. We present the outputs from the analyzed parameters in **Supplementary Table 1** (for drought) and **Supplementary Table 2** (for salinity), noting the Si and stress treatments that were applied and the strength of the reported effects (the ‘effect size’). We subsequently compared the measured variables for plants subjected to stress with and without Si treatment across all studies to produce average effect sizes which are depicted in **Figure 4**.

There are many examples of Si improving drought and salinity tolerance, both in Si-accumulating species such as rice (Wang et al., 2019; Yang et al., 2019) barley (Joudmand and Hajiboland, 2019), and wheat (Gong et al., 2003; Pei et al., 2010; Tale Ahmad and Haddad, 2011; Maghsoudi et al., 2016), and in ‘non-accumulators’ such as tomato (Cao et al., 2015; Shi et al., 2016). Si fertilization typically improves a wide variety of physiological parameters relating to drought and salinity stress, either completely or partially restoring them to levels observed in unstressed plants (Frew et al., 2018). We discuss the most prominent of these (**Figure 4**) below and evaluate their relevance.

### Oxidative Stress

Salinity and drought induce oxidative damage, a phenomenon that has been observed multiple times and is well established in the literature. Oxidative stress manifests itself as raised levels of ROS which can cause protein and lipid peroxidation, reduced membrane stability and consequent increased electrolyte leakage. Si fertilization often results in reduced oxidative damage and hence growth improvement. In our literature search, we found that under both drought and salinity stress, Si reduces oxidative damage by an average of ~30% (**Figure 4**). This is likely a result of increased antioxidant enzyme activity, which was increased an average of 20% under drought and 50% under salinity stress. Sattar et al. (2019) found that under drought stress, SOD, CAT, and POX activity increased in wheat but more so in the presence of Si. Similar observations were published by Kang et al. (2016) using the drought exposed xerophyte *Zygophyllum xanthoxylum*, by Saleh et al. (2017) using salt-treated wheat, and a host of other reports covering a multitude of plant species.

One of the few exceptions is the work by Gong et al. (2008) which found *decreased* CAT activity, and no difference in SOD or POD activity in drought exposed wheat. In support of the notion that Si augments (enzymatic) antioxidant activity, Si fertilization was also found to lower lipid peroxidation (Abbas et al., 2015; Soundararajan et al., 2017) and reduce electrolyte leakage (Tuna et al., 2008; Soleimannejad et al., 2019). In drought stressed sunflower, the effect of Si on antioxidant enzyme activity varied among cultivars, and only 8 of 12 cultivars tested had decreased H_2_O_2_ levels with Si (Gunes et al., 2008).

**Figure 2:**
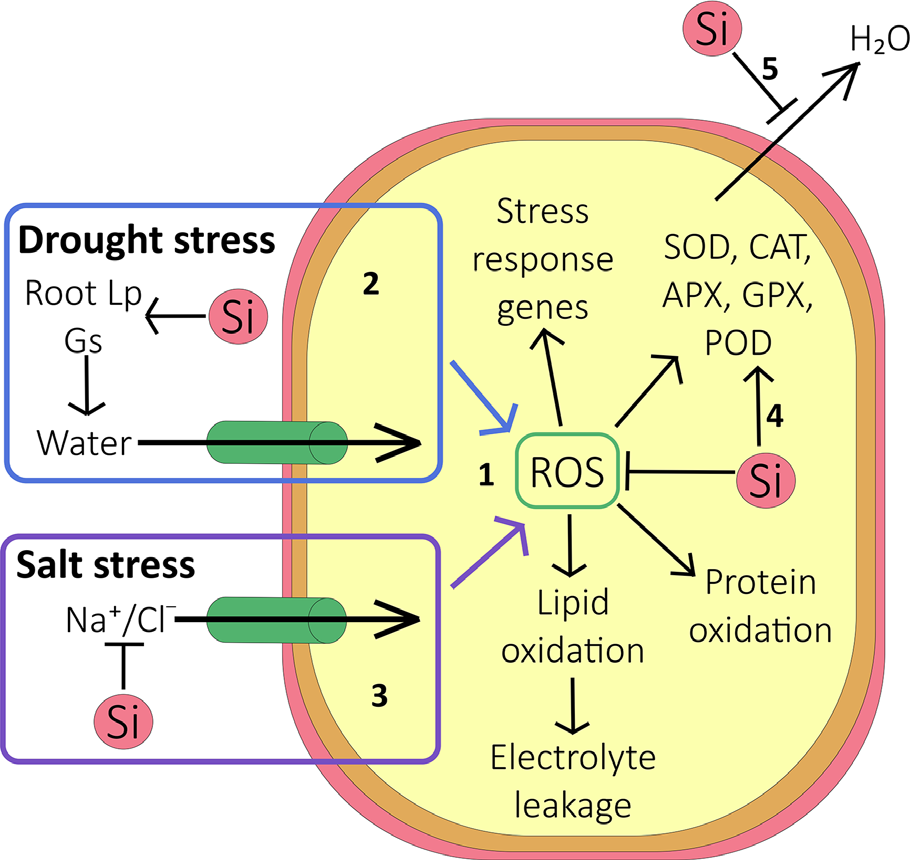
Effect of Si on oxidative stress. (1) Under abiotic stress conditions, accumulation of reactive oxygen species (ROS) inside the cell causes protein oxidation, lipid oxidation (resulting in increased electrolyte leakage out of the cell), and activation of stress response genes. (2) During drought stress, Si increases the root hydraulic conductance and stomatal conductance, but reduces cuticular transpiration (5). On balance this can allow more water to enter the cell and thus reduce the accumulation of ROS. (3) During salt stress, as well as improving the plant water status, Si reduces Na^+^ and Cl^-^ accumulation in shoot by forming endodermal barriers in the root. This reduces the accumulation of ROS and limits ion toxicity. (4) Antioxidative enzymes are activated by increased cellular ROS, and their activity may be further increased by Si. These enzymes scavenge ROS within the cell, thus protecting it against oxidative damage. (5) Si deposited outside the cell reduces evapotranspiration, protecting the plant against water stress.

The large number of studies, pertaining to a range of species, that purports reduced oxidative stress *via* improved enzyme activity, strongly suggests a link between the latter and Si (**Figure 2**). Further evidence comes from a report by Ma et al. (2016) that linked Si treatment to increased expression of oxidative stress genes. However, we are still completely in the dark where mechanistic models are concerned (**Figure 2**); increased enzyme activity could be achieved *via* transcriptional pathways or post transcriptionally. Either pathway would need molecular interactions taking place in the cytosol. It would also require some Si ‘sensor’ to set these pathways in motion. Given the overwhelmingly apoplastic location of Si, such mechanisms appear very unlikely and an indirect effect is more probable, for example Si induced alterations of water and ionic fluxes.

### Photosynthesis

Si fertilization can improve photosynthetic parameters under drought and salinity stress. Applying Si has been shown to increase the content of chlorophyll and other pigments in Si accumulators such as rice (Chen W. et al., 2011) and wheat (Maghsoudi et al., 2016; Sienkiewicz-Cholewa et al., 2018) but also in ‘non-accumulators’ such as tomato (Muneer et al., 2014; Cao et al., 2015) and tobacco (Hajiboland et al., 2017). Our analysis (**Figure 4**) found that increases in chlorophyll content was one of the largest effects of Si addition to salt stressed plants. Nevertheless, the increase in chlorophyll due to Si fertilization varies depending on the species and cultivar and it is not always significant (Mahdieh et al., 2015; Ali et al., 2018). Abbas et al. (2015), working with salt exposed okra, show that at least for this species, many of the Si-induced effects are not stress-specific and also occur in non-stressed plants. In sugarcane, only two of four cultivars measured showed increased chlorophyll in response to Si under drought stress (de Camargo et al., 2019).

Several models have been put forward to explain how Si could modify photosynthetic parameters; Si effects on stomatal function (see below) could impact on photosynthetic efficacy by altering gas exchange relations. Si fertilization often increases stomatal conductance (Sonobe et al., 2009; Kang et al., 2016; Sattar et al., 2019), and hence photosynthetic rates (Gong and Chen, 2012; Liu et al., 2014; Amin et al., 2018). Lower levels of oxidative stress will also promote photosynthesis *via* reduced pressure on the general cellular biochemistry, or by maintaining chloroplast and thylakoid integrity. More direct influence of Si on photosynthesis could be in the form of better allocation of light between the photosystems and hence a higher quantum efficiency (e.g. Cao et al., 2015), however, no mechanistic explanation of this phenomenon has been put forward.

### Nutrition

The influence of Si on nutrient content is highly variable. For Si itself, Cooke and Leishman (2016) found that among plants fertilized with Si, plant Si concentration is typically lower when considering all stresses they evaluated. However, no significant change was seen in plants treated with water stress while salinity stress led to a significant increase in shoot Si levels. There do not appear to be any consistent effects of Si on the content of other nutrients during drought or salinity exposure. Pei et al. (2010) found that Si decreased Mg, K, and Ca content in drought-stressed wheat. However, Bukhari et al. (2015), using the same species, reported that Si had no effect on Mg content and variable effects on K depending on the cultivar and application method used. The effects of Si on nutrient content under saline conditions are also varied; some studies mention Si-dependent increases in K uptake (e.g. Ali et al., 2012; Wang S. et al., 2015 in cucumber), a mechanism that would raise the tissue K^+^:Na^+^ ratio, whereas many others do not (e.g. Yin et al., 2016; Flam-Shepherd et al., 2018).

### Water Status

A large number of reports shows evidence that Si fertilization alters the water status of drought and salinity stressed plants (Gong and Chen, 2012; Sayed and Gadallah, 2014; Yin et al., 2014; Hasanuzzaman et al., 2018). As shown in **Figure 4**, this is partly a consequence of Si increasing water use efficiency (WUE), as has been reported in rice (Chen W. et al., 2011) and tobacco (Hajiboland et al., 2017), as well as increased levels of compatible solutes in Si fertilized plants (Pei et al., 2010; Sayed and Gadallah, 2014; Hajiboland et al., 2017; Yang et al., 2019). WUE appears to be modulated by Si in several ways, one of which is *via* transpiration. While Si deposited beneath the cuticle could reduce cuticular transpiration, and therefore limit water loss, this appears to be counteracted by the frequently observed increase in stomatal conductance (**Figure 4**). The latter could raise photosynthetic efficiency (see above) but is likely to lower WUE. Transpiration can also be manipulated by altering the root hydraulic conductance, for example by modulating aquaporin activity. In sorghum, Si application increased the expression at the transcript level and the activity of a number of aquaporins and thus counteracted the salt-induced lowering of the root hydraulic conductance (Liu et al., 2015). However, in tomato, no consistent changes in aquaporin gene expression were found as a result of Si fertilization (Shi et al., 2016).

Overall, Si appears to increase the transpiration rate (**Figure 4**), although this is variable between studies. Increased transpiration has been reported in drought- and salt-stressed rice and sorghum (e.g. Chen T. et al., 2011; Yin et al., 2014; Liu et al., 2015; Flam-Shepherd et al., 2018), although Yang et al. (2019) found that it took 3 weeks for Si to significantly increase the transpiration rate in drought-stressed rice. The effect of Si on transpiration can also vary between genotypes. In rice, while Si decreased transpiration in a salt-tolerant cultivar, the transpiration rate was increased in a salt-sensitive cultivar (Farooq et al., 2015).

Where compatible osmolytes are concerned, a similar confusing picture emerges with Si being reported to increase (Tale Ahmad and Haddad, 2011; Alzahrani et al., 2018) and decrease (Pei et al., 2010) proline levels in drought exposed wheat. Further studies, using other species, also reported both higher (Hajiboland et al., 2017) and lower (Sayed and Gadallah, 2014; Yin et al., 2014; Hasanuzzaman et al., 2018; Yang et al., 2019) proline levels in Si-treated plants. In several species, Si appears to increase the level of soluble sugars under salt and drought stress (rice: Yang et al., 2019; wheat: Alzahrani et al., 2018; okra: Abbas et al., 2015; tobacco: Hajiboland et al., 2017), however, Kang et al. (2016) reported decreased soluble sugar content in osmotically-stressed *Zygophyllum xanthoxylum*. Although in general Si appears to increase glycine betaine levels (Abbas et al., 2015; Saleh et al., 2017; Ahmad et al., 2019; Al-Huqail et al., 2019), decreases have been reported for drought-stressed maize (Parveen et al., 2019) and salt-stressed borage (Torabi et al., 2015). There is a trend for Si to increase polyamine levels under drought and salt-stress (Wang H. S. et al., 2015; Yin et al., 2016; Ali et al., 2018; Yin et al., 2019). In osmotically stressed tomato, different genotypes accumulated different amino acids in response to Si, although in both genotypes this resulted in increased polyamine accumulation (Ali et al., 2018). Overall, a small increase in compatible solutes due to Si fertilization was observed (**Figure 4**).

### Ion Transport

**Figure 3:**
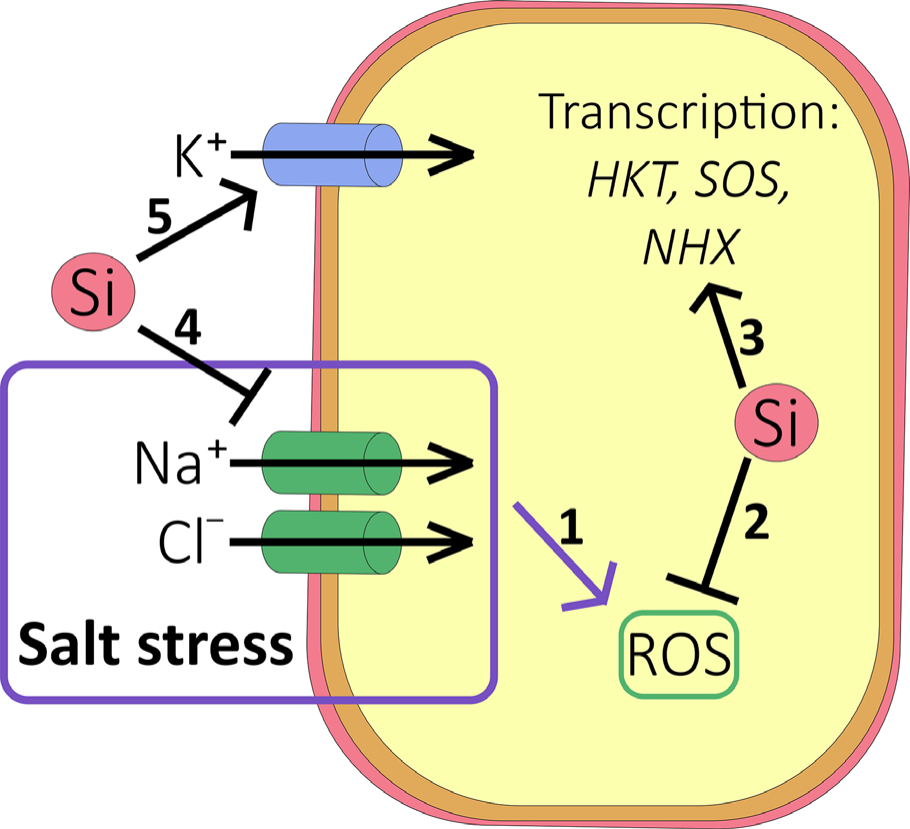
Effect of Si on salt accumulation. (1) Under salt stress conditions, accumulation of Na^+^ and Cl^-^ results in reactive oxygen species (ROS) accumulation and oxidative damage to the cell (see ). (2) Si inhibits the production of ROS in a number of ways (see ), protecting the cell against oxidative damage. (3) Si may increase the transcription of HKT1, SOS, and NHX transporters to relieve ion toxicity. (4) Si reduces root-to-shoot translocation of Na^+^ and Cl^-^. (5) Si may also stimulate accumulation of K^+^ into the cell to improve the K^+^:Na^+^ ratio.

The effects of Si on ion transport under drought or saline conditions are varied (**Figure 3**). According to several reports (Gong et al., 2006; Tahir et al., 2006; Tahir et al., 2010; Ali et al., 2012) Si treatment led to increased potaessium uptake which could alleviate salinity stress by improving the tissue K^+^:Na^+^ ratio while it provides cheap osmoticum to rebalance water relations during drought. However, further studies using other members of the Poacea family did not find a significant effect of Si on K content in either rice (e.g. Flam-Shepherd et al., 2018) or sorghum (e.g.Yin et al., 2016).

**Figure 4:**
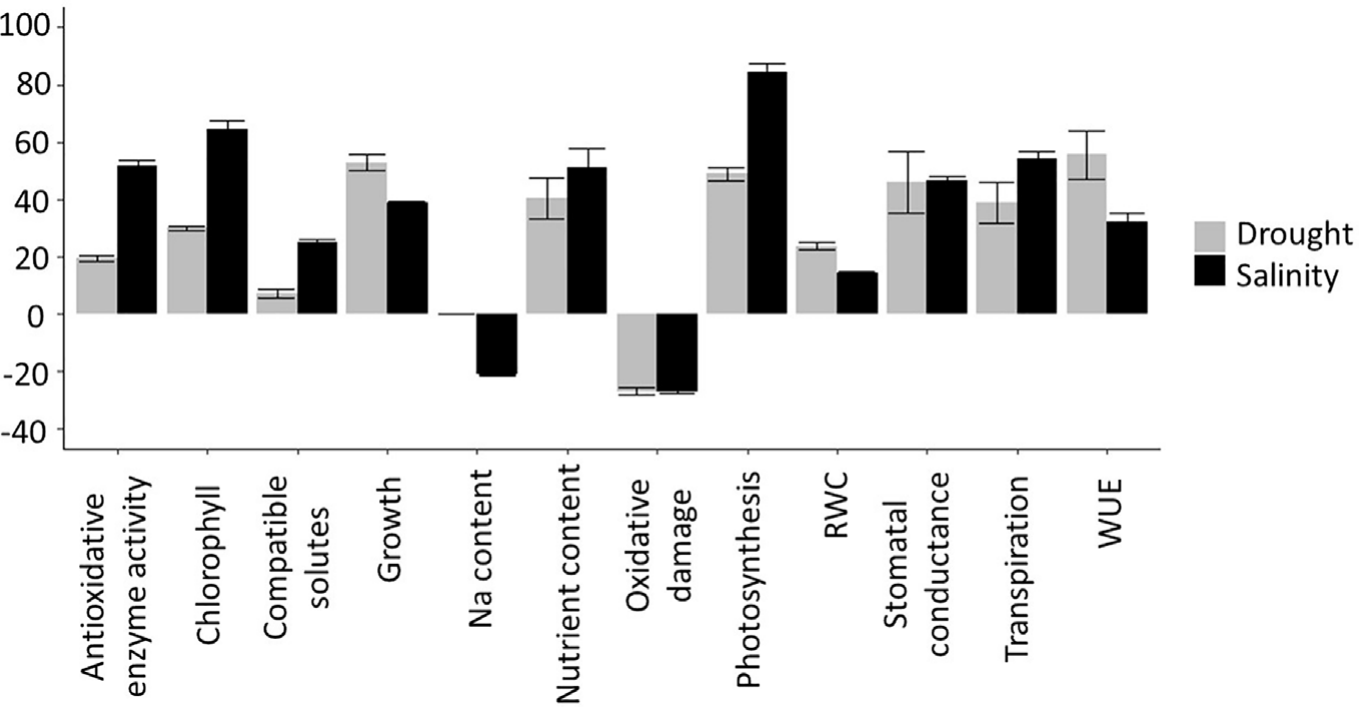
Summary of Si effects on drought and salinity stress. To determine the effect of Si treatment on each measured parameter, the effect size was calculated based on the reported measured values as: $\frac{Si+Stress-Stress}{Stress}\times 100\%$ Effect size values were then averaged across all publications that were included in the analysis (see and ) to produce an overall Si effect.

Ionic toxicity due to salinity mainly stems from excess Na^+^ and Cl^-^ ions and is significantly alleviated by Si fertilization (**Figures 3** and **4**). Accumulation of Na^+^ (and Cl^-^) in the shoot (but not the root) has been shown to be consistently lower in most species tested so far, including high and low Si accumulators. Examples of studies supporting this result include Ahmad et al. (1992); Muneer et al. (2014); Abbas et al. (2015) and Yin et al. (2016) and a meta-analysis that looked across 18 different species (Cooke and Leishman, 2016). Nevertheless, this may need some qualification since the Si-induced effect may be temporarily as reported by for example Chen et al. (2014) who showed the Na^+^ concentration was lower in Si fertilized wheat for the first 20 days of stress only. As the duration of salt stress increased, the leaf Na^+^ level became higher in Si fertilized plants (Chen et al., 2014). Likewise, Bosnic et al. (2018) reported that the Na^+^ concentration of Si-treated maize was higher than that found in Si-untreated plants after 14 days of salt stress, with Na^+^ being sequestered into the vacuoles. In contrast, Khan et al. (2018) showed reduced tissue levels of Na^+^ in maize but the effect depended on both genotype and the plant organ and similar observations are plentiful in the literature (**Supplementary Table 2**).

The mechanism by which Si reduces ion transfer to shoot tissue has been studied relatively well, particularly for Na^+^, by using apoplastic tracers (e.g. Gong et al., 2006). In salt-stressed rice for example, Si blocks apoplastic bypass flow in the root which occurs in regions where the Casparian strip is incomplete. Blockage may involve the polymerization of silicic acid within the endodermal apoplast, for example *via* complexation with lignin and other phenolics. Results from material science studies show that SiO_2_ can covalently bind to lignin (e.g. Strzemiecka et al., 2016) an idea that is strengthened by NMR studies on lignin-silica co-precipitates (Cabrera et al., 2016). The ensuing physical barrier will limit both ion and water permeability, forcing a relatively large proportion to move *via* the symplast where flux control is far greater. Alternatively, Si could promote suberisation and lignification of the Casparian strip itself, for example by altering transcript levels of relevant genes (e.g. Hinrichs et al., 2017).

Interestingly, a tightening of the Casparian strip would not only restrict ion flux but also the water flux and hence lower transpiration rates (as has been reported numerous times for salinised plants). Notwithstanding, many authors report on a relative *increase* in transpiration after Si treatment of salinised plants (e.g Liu et al., 2015; Wang H. S. et al., 2015; Manivannan et al., 2016) as shown in **Figure 4**. This indicates that manipulation of other parts of the transpirational pathway by Si, such as enhancing the stomatal conductance, can partially compensate the overall reduction in water flux.

### Correlation Between Effect Size and Tissue Si

Of the 21 studies in **Supplementary Tables 1** and **2** that compared multiple cultivars, very few contain data on plant Si content. In sugarcane, no correlation between Si content and either dry weight, RWC, or pigment content was found in drought stressed plants (de Camargo et al., 2019). For rice, one study showed the sensitive cultivar having a higher Si content (Farooq et al., 2015), while the opposite was reported in another (Mahdieh et al., 2015), nevertheless, in neither study did the cultivar with the highest Si content show a bigger Si effect. In chickpea, the tolerant cultivar had a higher Si content than the sensitive cultivar and showed a bigger Si effect in all the parameters that were measured (Garg and Bhandari, 2016a; Garg and Bhandari, 2016b). Similarly, in okra, the tolerant cultivar had a higher root Si content, and showed a bigger Si effect for most measured parameters (Abbas et al., 2015). More detailed studies are urgently needed to establish if and how Si effects relate to tissue levels, especially with the aim to inform accurate cost-benefit analyses (see below).

## Contrasting Results From Omics Studies

Recently, there has been interest in better understanding the mechanisms underpinning the benefits of Si by using transcriptomic, proteomic, and metabolomic approaches.

### Transcriptomics

Many studies have proposed that Si fertilization can affect gene expression (reviewed in Manivannan and Ahn, 2017). For example, ambient Si levels influence expression of both *Lsi1* and *Lsi2* with Lin et al. (2019) reporting a modest upregulation of around 2-fold while Yamaji et al. (2008) and Mitani-Ueno et al. (2016) reported down regulation when external Si was increased. The latter identified a -327 to -292 promoter region involved in this regulation.

In general, Si fertilization does not appear to significantly affect gene expression when applying control (non-stress) treatment (Watanabe et al., 2004; Chain et al., 2009). The small number and lack of consistency of transcripts affected by Si under control conditions, is in agreement with the idea that Si has little effect on plants in those settings (e.g. Cooke and Leishman, 2016). A seemingly conflicting study was provided by Holz et al. (2015) who found 572 up- and 564 downregulated cucumber genes when Si was supplied in control conditions. Likewise, Zhu et al. (2019) identified 1237 up- and 232 downregulated genes when Si was supplied to cucumber grown in control conditions, which were mainly related to the plant stress response, metabolism, signaling, and ion homeostasis. Brunings et al. (2009) identified 105 up- and 116 downregulated genes between control and Si-treated plants, which were mainly housekeeping genes or those involved in the defense response.

To date, few transcriptomic studies have examined the effects of Si under salt or drought stress. Zhu et al. (2019) conducted a transcriptome study regarding the effect of Si on cucumber under salt stress and found only 19 genes up- and 10 genes downregulated between salt stressed plants supplied with Si and salt stressed plants without Si. However, when comparing salt-stressed plants treated with and without Si to plants grown under control conditions, it was found that applying Si reduced both the increase in expression of upregulated genes and the decrease in expression of downregulated genes. Thus, overall, applying Si to salt-stressed cucumber acted to bring the transcriptome back to be more similar to that seen in control conditions (Zhu et al., 2019). Transcriptomic studies examining the effects of Si under pathogen stress have reported similar abilities of Si to restore the transcriptome to more closely resemble that observed under control conditions (Fauteux et al., 2006; Chain et al., 2009).

In maize, Si fertilization of salt treated (40 mM NaCl, 14 days) plants was linked to increased *SOS1* and *SOS2* expression, decreased *HKT1* expression in the roots and increased *NHX* expression in the leaf (Bosnic et al., 2018). This paper ‘bucks the trend’ of Si-induced reductions in shoot Na^+^ levels (e.g. Khan et al., 2018 for maize) and reported *increased* levels of leaf Na^+^. This makes it most likely that the changes in transcript level are due to altered Na^+^ fluxes and concentrations, rather than a direct effect of Si, since there is good evidence for salt induced transcriptional regulation of *SOS, HKT*, and *NHX*.

### Proteomics

Protein expression does not necessarily mirror transcript levels and generally has a greater functional relevance than gene expression. By comparing Si-fertilized and non-fertilized rice, Jang et al. (2018) identified only 7 Si-regulated proteins after 12h, and these were involved in diverse functions. In tomato, semi-quantitative thylakoid proteomics showed that Si treatment limited the salt-induced loss of photosystems (Muneer et al., 2014 5-day treatment). In rose, Si addition was also found to restore the proteome of salt-stressed plants similar to that observed under control conditions (Soundararajan et al., 2018 15-day treatment).

In barley, Si has been shown to increase H^+^-ATPase activity (Liang et al., 2006). This system is responsible for setting up a proton motive force to drive secondary transporters that mediate Na^+^ efflux and hence contribute to salt tolerance. The authors followed the ATPase activity during a time course of 0, 2, 4, and 6 days and only reported significant differences after 4 and 6 days. However, similar experiments in rice did not generate corroborating results (Coskun et al., 2016).

### Metabolomics

Few studies have extensively examined the effect of Si on the plant metabolome. In a partial metabolomic study in rice, Si was found to stimulate amino acid remobilization (Detmann et al., 2012). In cowpea, Si-treatment resulted in only minor changes to the metabolome (L, 2012). In date palm, 41 metabolites were identified in the leaves and 54 in the roots of salt-stressed Si-treated plants that were not found in plants that did not receive Si. Additionally, 12 metabolites were found only in the leaves and 17 only in the roots of Si-treated salt-stressed plants. Many of the metabolites were antioxidants or osmoregulators and it was argued that Si promoted detoxification pathways (Jana et al., 2019).

## GM to Increase Plant Si Accumulation

On balance, Si treatment significantly improves tolerance to drought and salinity (**Supplementary Tables 1** and **2**; **Figure 4**). This suggests that the use of GM to increase Si accumulation may be a promising strategy to develop more resilient crops. Though studies focussing on salinity or drought are lacking, work in rice has shown that over-expression of *OsLsi1* can be used to increase Si accumulation, and this is correlated with improved cold-stress tolerance (Azeem et al., 2016; Fang et al., 2017) and UV-B tolerance (Fang et al., 2011). Interestingly, the constitutive expression of *TaLsi1* or *OsLsi1* in the non-accumulator Arabidopsis increased Si accumulation but caused deleterious effects (Montpetit et al., 2012). After use of a root specific promoter the increased Si uptake capacity was retained but no improvements in stress tolerance were reported (Montpetit et al., 2012). In tomato, another Si non-accumulator, transformation with *CsLsi2* increased Si accumulation and improved heat and water stress tolerance (Sun et al., 2020). Transformation of Indica rice with *OsLsi1* from Japonica rice increased Si accumulation, antioxidative enzyme activity, and chlorophyll content (Sahebi et al., 2017).

## Agronomic Feasibility of Si Fertilization—A Cost-Benefit Analysis

Si supply is not limited, has no known environmental downsides and the above discussion shows that Si fertilization can be a very effective strategy to increase crop production in areas that are compromised by abiotic stresses such as drought and salinity. Although Si itself is plentiful, the bioavailable fraction is small and since 7 of 10 most grown crops are Si-accumulators, the potential exists to extract large amounts of bioavailable Si from the soil (Ma and Yamaji, 2006; Guntzer et al., 2012). Many soils therefore are, or risk being, Si deficient and require Si supplements.

Evaluation of the efficacy of Si fertilization in an agronomic context must consider the economic feasibility. Si fertilizer comes in several forms: Steel making and blast furnace slags, Na-silicate (Na_2_O_3_Si), K-silicate (K_2_O_3_Si) and Ca-silicate (CaO_3_Si or Wollastonite). Slags typically contain low levels of Si (10%–15%; Ito, 2015) and are relatively cheap (€20–35/t, equivalent to €140–350/t Si). However, they contain a large number of additional nutrients (Ca, K, N, P; Ito, 2015) and potentially toxic metals, in particular Al and Fe. Thus, it is near impossible to evaluate the specific Si effects when using these fertilizers and they are therefore not considered any further here. Wholesaler supplied Na- or K-silicate typically costs €140–200/t (https://www.alibaba.com/showroom/sodium+silicate+price.html) and contain ~23 and ~18% Si respectively which translates to €600–1,050/t Si. Wollastonite has a comparable Si content (~24%) to that of Na-silicate and is somewhat cheaper, working out at €350–600/t Si (https://www.alibaba.com/showroom/wollastonite-price.html).

To achieve sustainability, the Si offtake due to crop harvesting has to be compensated by Si input. Input can originate from the soil solution (which in turn is fed by weathering of soil minerals and contributions from irrigation practices), from crop residues and from fertilization. Assuming the latter is the only form of Si replenishment, a yield of 4t/ha, and a crop Si concentration of 5% (DW), 200 kg/ha Si is required to compensate for offtake, adding a cost of approximately €80–180/ha. Whether this extra cost makes economic sense will depend on a large number of factors such as crop type, yield per hectare, anticipated yield gain and production costs. For example, in many SE Asian countries average paddy rice yield is ~4t/ha with production costs that are typically €600–1,200/ha (Liese et al., 2014). In this scenario, an extra €80–180 would add 6%–27%, extra input costs that would constitute a sensible investment only if significant gains in yield (>10%) can be expected. In comparison, yield in countries like Korea, Japan and the USA (USDA, 2019) are much higher (7–8 t/ha) requiring larger Si inputs of 350–400 kg/ha. But with considerably higher production costs of €3,000–6,000/ha in Japan (Food and Fertilizer Technology Center, 2009) and €2,500–3,500/ha in the USA (USDA, 2019) the relative extra cost (€130–340) adds only around 3%–17%. In other words, farmers in areas where production costs are low, as is typically the case in developing countries, would need to see a larger improvement in crop yield to justify *a priori* expenditure in the form of fertilizer application.

The above calculation ignores additional benefits that could stem from unrelated effects such as reduced lodging or improved pathogen resistance. Furthermore, it is based on rice (a very strong Si accumulator) and with Si fertilizer as the sole input to replenish offtake. In most soils, the solution contains plant-available Si which usually ranges between 0.1–1.0 mM (Epstein, 1994 PNAS; Pradeep et al., 2016). Assuming a depth of 50 cm, this soil solution reservoir could provide 15–150 kg of plant available Si and thus decrease reliance on Si supplement. Phytoliths that are located mainly in shoots of monocots can return to the soil through litterfall and so contribute to the biogeochemical cycle of Si (Alexandre et al., 1997). Recycling of plant material such as plowing back cereal straw can therefore be another substantial contributor to Si sustainability. For rice, straw makes up 40%–50% of the total biomass and straw contains far higher levels of Si than roots and panicles (Agostinho et al., 2017). Thus, rice straw recycling would provide more than half the necessary Si supplementation calculated above. A further issue to consider is the necessity of replacing *all* Si offtake; studies on the critical tissue levels of Si are often lacking as are evaluations of Si fertilizer efficacy.

The cost benefit analysis also ignores a number of potential drawbacks where the use of Si fertilizer is concerned. Sporadic publications report on negative impact of Si on biomass and seed production as exemplified by studies on *Glycyrrhiza uralensis* (Zhang et al., 2017), *Zygophyllum xanthoxylum* (Kang et al., 2016), strawberry (Dehghanipoodeh et al., 2018) and pepper (Trejo-Téllez et al., 2020). Furthermore, cheaper forms of Si fertilizer can be contaminated with toxic metals which will slowly build up in the soil and could create future yield loss and health issues. Another drawback is the negative effect of Si on digestibility; livestock tends to avoid fodder with high Si content such as rice straw, forcing farmers to burn crop residues. High Si content also reduces potential recycling of crop residues in the form of biofuel. This is the case because tissue digestion, as part of the fermentation process, is slower in the presence of high Si levels. If tissue is used for direct combustion, tissue Si reacts with alkali elements to form slag deposition in furnaces. Although modest when compared to nitrogen fertilizer production, mining and transport of Si fertilizer (e.g. as Wollastonite) has a carbon footprint that contributes to global warming. The presence of Si can also impact on rhizosphere microbial communities and hence the decomposition of organic materials. For example, it was shown that Si delays leaf litter decomposition in reeds because it limited growth of fungal decomposers (Schaller et al., 2014).

## Conclusions and Outlook

Overall, Si fertilization has been found to have many beneficial effects in plants under drought and salt stress. The investigated physiological parameters in these studies varied considerably and the effects of Si upon them greatly depended on plant species, genotype, growth stage, and stress severity. For both drought and salinity, the parameters that showed the largest positive effects were photosynthesis and chlorophyll levels (**Figure 4**). Si also shows a consistent reduction in oxidative damage in a variety of crops and conditions, which correlates with a significant increase in the activity of antioxidant enzymes (although no consistent effect on the expression of antioxidative enzymes was found). In parallel, there is very convincing evidence that Si is predominantly located in the apoplast where it can help form physical barriers and mechanical strength, for example in the root endodermis or leaf epidermal cell wall (Luyckx et al., 2017; Coskun et al., 2019). The concomitant alteration of water and ion fluxes could in turn explain how Si alters generic processes such as oxidative stress or photosynthesis. Other reported benefits of Si, such as reduced lodging or protection against herbivores, would fit in well with this model.

A key question remains whether Si functions purely at the physico-chemical level, for example *via* its deposition in root and shoot cell walls that causes altered water and ion fluxes, or that Si interacts with intracellular processes such as gene expression. As yet, the evidence for such a “biochemical” role of Si is far less convincing; studies report very contradictory outcomes and generally very low numbers of genes/proteins that are altered by Si treatment. Methodological problems include the long periods of treatment (days or even weeks) after which changes in transcript/protein levels are measured which greatly increases the probability that secondary effects are observed, for example *via* altered ion and water fluxes. In many studies, K, Na or Ca silicate are used as Si source and unless proper control experiments are carried out, these silicates increase cation concentrations and thus can severely alter plant nutrition. Hard evidence that Si directly influences gene transcription will need reproducible, short time scale (minutes to hours) studies. The use of more tractable systems such as cell cultures would also help greatly to settle this issue. Convincing evidence of the involvement of specific genes and proteins could then be extended with mutational studies to unravel any putative biochemical role for Si.

Our limited analysis shows a positive cost benefit when yield gains of more than around 10% can be expected. Although not the same as yield in an agronomic sense, gains in biomass of 10% or more have been described frequently in laboratory settings (e.g. Guntzer et al., 2012). Effect magnitudes are sensitive to species and cultivar variation, implying that analyses of critical Si levels in soils and in plant tissues that are necessary to maximize yield gain will need to be carried out multiple times. Thus, there may be many regions and climatological conditions where agriculture can profit from increased Si fertilization but more research is needed, particularly focussing on the long-term benefits of Si under field conditions.

## Data Availability Statement

The datasets presented in this study can be found in online repositories. The names of the repository/repositories and accession number(s) can be found in the article/supplementary material.

## Author Contributions

All three authors analyzed literature data and wrote manuscript.

## Funding

BBSRC funded a 4y scholarship to ST, ref 1949569.

## Conflict of Interest

The authors declare that the research was conducted in the absence of any commercial or financial relationships that could be construed as a potential conflict of interest.
