# Supplementary material for: Is Silicon a Panacea for Alleviating Drought and Salt Stress in Crops?
Source: Front Plant Sci. 2020 Aug 18;11:1221. doi: 10.3389/fpls.2020.01221 (PMC7461962; doi:10.3389/fpls.2020.01221)
Supplement: Supplementary file 1 [file Table_1.docx]

**Table 1: The impact of Silicon on plant Drought Stress**

A literature search was carried out to evaluate how Si affects plant drought stress (see * for used criteria). The observed impacts of various parameters were classified from ‘non-significant’ to ‘very large’ (see **). Where multiple Si or stress treatments were used, data are reported for the most beneficial dose of Si and for the highest stress level only. The term ‘hydroponics’ was used to describe any study where the main source of nutrients was from a nutrient solution (e.g. includes studies using sand/perlite).

Abbreviations: relative water content (RWC); water use efficiency (WUE); days after sowing (DAS); field capacity (FC); water holding capacity (WHC)

| **Measured variable** | **Species** | **Reference** | **No. citations** | **Level of Si** | **Level of stress** | **Length of stress** | **Harvest growth stage** | **Growth conditions** | **Effects on growth (under stress)** | **Strength of effect** |
| --- | --- | --- | --- | --- | --- | --- | --- | --- | --- | --- |
| Oxidative damage | Wheat | Ma *et al*. (2016) | 36 | 204 mg kg^-1^ Na silicate | 55% RWC | 10 and 20 days | 10 and 20 days after heading | Soil | Unknown | 10 d: – –  20 d: – – |
|  | Tomato | Shi *et al*. (2016) | 36 | 2.5 mM K silicate | 10% PEG-6000 | 7 days | 7 days after 5-leaf stage | Hydroponics | + + + + | – – – |
|  | Tomato | Cao *et al*. (2015) | 21 | 0.6 mM Na silicate | 1 % PEG-6000 | 12 days | 12 days after 9-leaf stage | Hydroponics | + + + | – – |
|  | Wheat | Alzahrani *et al*. (2018) | 20 | 4 mM K silicate | 50% FC | 45 days | 60 days | Soil | + + + | – – – |
|  | Rapeseed | Hasanuzzaman *et al*. (2018) | 16 | 1 mM SiO_2_ | 20 % PEG | 48 hours | 14 days | Hydroponics | Unknown | – – – |
|  | Cowpea | Merwad *et al*. (2018) | 13 | 2 mM K silicate (foliar spray 15, 30, 45 DAS) | 20 % WHC | 55 and 80 days | 80 days | Soil | + + | – – |
|  | Cucumber | Ouzounidou *et al*. (2016) | 12 | 2.25 mM K silicate (foliar spray every 10 days) | Irrigation reduced by 50% | 3 months | When fruits ripe | Soil | Sensitive: + + +  Tolerant: + + + + | Both cultivars: – – – |
|  | Barley | Maillard *et al*. (2018) | 8 | 1.5 mM silicic acid | 190 g L^-1^ PEG-6000 | 4 days | 2 weeks | Hydroponics | No effect | No effect |
|  | Strawberry | Moradtalab *et al*. (2019) | 2 | 0.6 mM Na silicate | 35% WHC | 4 weeks | 6 weeks | Hydroponics | + + + | – – – |
|  | Maize | Parveen *et al*. (2019) | 0 | 6 mM Na silicate | 60% FC | 3 weeks | 1 month | Soil | Both cultivars: + + + + + | Both cultivars: – – – – |
| Anti-oxidative enzyme activity | Tomato | Shi *et al*. (2016) | 36 | 2.5 mM K silicate | 10% PEG-6000 | 7 days | 7 days after 5^th^ leaf stage | Hydroponics | + + + + | + + + + |
|  | Tomato | Cao *et al*. (2015) | 21 | 0.6 mM Na silicate | 1 % PEG-6000 | 12 days | 12 days after 9-leaf stage | Hydroponics | + + + | – – |
|  | Wheat | Alzahrani *et al*. (2018) | 20 | 4 mM K silicate | 50% FC | 45 days | 60 days | Soil | + + + | + + + + + |
|  | Rapeseed | Hasanuzzaman *et al*. (2018) | 16 | 1 mM SiO_2_ | 20 % PEG | 48 hours | 14 days | Hydroponics | Unknown | + + + + |
|  | Mango | Helaly *et al*. (2017) | 14 | 1.5 mM K silicate | Water potential of -0.77 bars | 3 years | 23 years | Soil | All cultivars: + + + + + | All cultivars: + + + |
|  | *Zygophyllum xanthoxylum* | Kang *et al*. (2016) | 12 | 2.5  mM K silicate | 30% FC | 10 days | 5 weeks | Vermiculture | + + + + | + + + + + |
|  | Strawberry | Moradtalab *et al*. (2019) | 2 | 0.6 mM Na silicate | 35% WHC | 4 weeks | 6 weeks | Hydroponics | + + + | + + + + + |
|  | Wheat | Maghsoudi *et al*. (2019) | 0 | 6 mM Na silicate (foliar spray, tillering and anthesis) | 40% FC | Anthesis to grain filling and ripening | Ripening | Soil | Unknown | 4 cultivars: generally + + + + + |
|  | Maize | Parveen *et al*. (2019) | 0 | 6 mM Na silicate | 60% FC | 3 weeks | 1 month | Soil | Both cultivars: + + + + + | Both cultivars: + + + + + |
|  | Sunflower | Seleiman *et al*. (2019) | 0 | 10 t ha^-1^ biochar applied before sowing or 150 g ha^-1^ K silicate 30 and 55 DAS | 90% water deficit | From sowing until flowering | Flowering | Soil | Unknown | Biochar: – – –  Silicate: – – |
| Anti-oxidant content | Wheat | Ma *et al*. (2016) | 36 | 204 mg kg^-1^ Na silicate | 55% RWC | 10 and 20 days | 10 and 20 days after heading | Soil | Unknown | 10 d: + + + + +  20 d: + + + + |
|  | Mango | Helaly *et al*. (2017) | 14 | 1.5 mM K silicate | Water potential of -0.77 bars | 3 years | 23 years | Soil | All cultivars: + + + + + | All cultivars: n.s. |
|  | Tomato | Ali *et al*. (2018) | 4 | 1.5 mM silicic acid | 1% PEG-6000 | 3 weeks | 4 weeks | Hydroponics | Sensitive: + + +  Tolerant: n.s. | Sensitive: n.s.  Tolerant: + + + + |
| Compatible solutes | Wheat | Alzahrani *et al*. (2018) | 20 | 4 mM K silicate | 50% FC | 45 days | 60 days | Soil | + + + | + + + + + |
|  | Mango | Helaly *et al*. (2017) | 14 | 1.5 mM K silicate | Water potential of -0.77 bars | 3 years | 23 years | Soil | All cultivars: + + + + + | All cultivars: + + + + + |
|  | Barley | Hosseini *et al*. (2017) | 13 | 1 mM Na silicate | 190 g L^-1^ PEG-6000 | 5 days | 26 days | Hydroponics | N.s. | N.s. |
|  | *Zygophyllum xanthoxylum* | Kang *et al*. (2016) | 12 | 2.5  mM K silicate | 30% FC | After 4 weeks, for 10 days | ~5 weeks | Vermiculture | + + + + | – – – |
|  | Cucumber | Ouzounidou *et al*. (2016) | 12 | 2.25 mM K silicate (foliar spray every 10 days) | Irrigation reduced by 50% | ~3 months | When fruits ripe | Soil | Sensitive: + + +  Tolerant: + + + + | Both cultivars: – – – |
|  | Barley | Maillard *et al*. (2018) | 8 | 1.5 mM silicic acid | 190 g L^-1^ PEG-6000 | 4 days | 2 weeks | Hydroponics | N.s. | N.s. |
|  | Strawberry | Dehghanipoodeh *et al*. (2018) | 4 | 10 mM K silicate | 0.75 soil moisture depletion | 3 months | 7 months | 1:1:1 Soil:Peat:Perlite | + + + | + + + + |
|  | Strawberry | Moradtalab *et al*. (2019) | 2 | 0.6 mM Na silicate | 35% WHC | 4 weeks | 6 weeks | Hydroponics | + + + | Shoot: – – –  Root: + + + + + |
|  | Rice | Yang *et al*. (2019) | 1 | 4000 mg kg^-1^ Ca silicate slag | 50% FC | 28 days | 56 days | Soil | + + | + + + + |
|  | Wheat | Maghsoudi *et al*. (2019) | 0 | 6 mM Na silicate (foliar spray, tillering and anthesis) | 40% FC | Anthesis to grain filling and ripening | Ripening | Soil | Unknown | Sensitive: + + +  Tolerant: + + |
|  | Maize | Parveen *et al*. (2019) | 0 | 6 mM Na silicate | 60% FC | 3 weeks | 1 month | Soil | Both cultivars: + + + + + | Both cultivars: – – – – – |
|  | Sunflower | Seleiman *et al*. (2019) | 0 | 10 t ha^-1^ biochar applied before sowing or 150 g ha^-1^ K silicate 30 and 55 DAS | 90% water deficit | From sowing until flowering | Flowering | Soil | Unknown | Biochar: – –  Silicate: – |
| Nutrient content | Wheat | Alzahrani *et al*. (2018) | 20 | 4 mM K silicate | 50% FC | 45 days | 60 days | Soil | + + + | + + + |
|  | Mango | Helaly *et al*. (2017) | 14 | 1.5 mM K silicate | Water potential of -0.77 bars | 3 years | 23 years | Soil | All cultivars: + + + + + | All cultivars: + + + + + |
|  | Cowpea | Merwad *et al*. (2018) | 13 | 2 mM K silicate (foliar spray 15, 30, 45 DAS) | 20 % WHC | 55 and 80 days | 80 days | Soil | + + | + + |
|  | Strawberry | Dehghanipoodeh *et al*. (2018) | 4 | 10 mM K silicate | 0.75 soil moisture depletion | 3 months | 7 months | 1:1:1 Soil:Peat:Perlite | + + + | – – – – |
|  | Strawberry | Moradtalab *et al*. (2019) | 2 | 0.6 mM Na silicate | 35% WHC | 4 weeks | 6 weeks | Hydroponics | + + + | N.s. |
|  | Rice | Ibrahim *et al*. (2018) | 1 | 560 mg L^-1^ K silicate (foliar spray 30, 40, 50, 60 DAS) | 70% soil saturation point | 2 ½ months | 3 ½ months | Soil | + + + + | + + + + |
|  | Wheat | Maghsoudi *et al*. (2019) | 0 | 6 mM Na silicate (foliar spray, tillering and anthesis) | 40% FC | Anthesis to grain filling and ripening | Ripening | Soil | Unknown | All cultivars: n.s. |
| Relative water content | Tomato | Shi *et al*. (2016) | 36 | 2.5 mM K silicate | 10% PEG-6000 | 7 days | 7 days after 5-leaf stage | Hydroponics | + + + + | + + + |
|  | Wheat | Alzahrani *et al*. (2018) | 20 | 4 mM K silicate | 50% FC | 45 days | 60 days | Soil | + + + | + + + |
|  | Durum wheat | Meunier *et al*. (2017) | 17 | 1.5 mM K silicate | 12 % PEG | 30 days | 35 days | Hydroponics | + + + + | + + + + |
|  | Rapeseed | Hasanuzzaman *et al*. (2018) | 16 | 1 mM SiO_2_ | 20 % PEG | 48 hours | 14 days | Hydroponics | Unknown | + + + |
|  | Mango | Helaly *et al*. (2017) | 14 | 1.5 mM K silicate | Water potential of -0.77 bars | 3 years | 23 years | Soil | All cultivars: + + + + + | All cultivars: + + + + |
|  | Barley | Maillard *et al*. (2018) | 8 | 1.5 mM silicic acid | 190 g L^-1^ PEG-6000 | 4 days | 2 weeks | Hydroponics | No effect | + + |
|  | Strawberry | Moradtalab *et al*. (2019) | 2 | 0.6 mM Na silicate | 35% WHC | 4 weeks | 6 weeks | Hydroponics | + + + | + + |
|  | Sugarcane | de Camargo *et al*. (2019) | 0 | 600 kg ha^-1^ Ca Mg silicate | 55% FC | 28 d | 6 months | Soil | + + + + | + + + + |
|  | Sunflower | Seleiman *et al*. (2019) | 0 | 10 t ha^-1^ biochar applied before sowing or 150 g ha^-1^ K silicate 30 and 55 DAS | 90% water deficit | From sowing until flowering | Flowering | Soil | Unknown | Biochar and silicate: + + + |
| Water use efficiency | Wheat | Alzahrani *et al*. (2018) | 20 | 4 mM K silicate | 50% FC | 45 days | 60 days | Soil | + + + | + + + |
|  | Cowpea | Merwad *et al*. (2018) | 13 | 2 mM K silicate (foliar spray 15, 30, 45 DAS) | 20 % WHC | 55 and 80 days | 80 days | Soil | + + | + + |
|  | *Zygophyllum xanthoxylum* | Kang *et al*. (2016) | 12 | 2.5  mM K silicate | 30% FC | 10 days | 5 weeks | Vermiculture | + + + + | + + + + |
|  | Cucumber | Ouzounidou *et al*. (2016) | 12 | 2.25 mM K silicate (foliar spray every 10 d) | Irrigation reduced by 50% | ~3 months | When fruits ripe | Soil | Sensitive: + + +  Tolerant: + + + + | Sensitive: + +  Tolerant: no effect |
|  | Wheat | Maghsoudi *et al*. (2015) | 11 | 6 mM Na silicate (foliar spray 30 DAS) | 40% FC | 20 days | 45 days | Soil | Tolerant: + + + +  Sensitive: + + +  (2 cultivars of each) | Tolerant: + + +  Sensitive: + + |
|  | Strawberry | Dehghanipoodeh *et al*. (2018) | 4 | 10 mM K silicate | 0.75 soil moisture depletion | 3 months | 7 months | 1:1:1 Soil:Peat:Perlite | + + + | + + + + + |
|  | Strawberry | Moradtalab *et al*. (2019) | 2 | 0.6 mM Na silicate | 35% WHC | 4 weeks | 6 weeks | Hydroponics | + + + | + + |
|  | Rice | Ibrahim *et al*. (2018) | 1 | 560 mg L^-1^ K silicate (foliar spray 30, 40, 50, 60 DAS) | 70% soil saturation point | 2 ½ months | 3 ½ months | Soil | + + + + | + + + + + |
| Transpiration | Tomato | Shi *et al*. (2016) | 36 | 2.5 mM K silicate | 10% PEG-6000 | 7 days | 7 days after 5-leaf stage | Hydroponics | + + + + | + + + |
|  | Wheat | Alzahrani *et al*. (2018) | 20 | 4 mM K silicate | 50% FC | 45 days | 60 days | Soil | + + + | + + + |
|  | Cucumber | Ouzounidou *et al*. (2016) | 12 | 2.25 mM K silicate (foliar spray every 10 days) | Irrigation reduced by 50% | 3 months | When fruits ripe | Soil | Sensitive: + + +  Tolerant: + + + + | Both cultivars: + + + + + |
|  | Maize | Amin *et al*. (2018) | 7 | 100 mg kg^-1^ Ca silicate | 60 % FC | 3 weeks after germination until maturity | Maturity | Soil | Both cultivars: + + + | 45 and 70: – – – – – |
|  | Strawberry | Dehghanipoodeh *et al*. (2018) | 4 | 10 mM K silicate | 0.75 soil moisture depletion | 3 months | 7 months | 1:1:1 Soil:Peat:Perlite | + + + | – – – – – |
|  | Strawberry | Moradtalab *et al*. (2019) | 2 | 0.6 mM Na silicate | 35% WHC | 4 weeks | 6 weeks | Hydroponics | + + + | + + + |
|  | Rice | Yang *et al*. (2019) | 1 | 4000 mg kg^-1^ Ca silicate slag | 50% FC | 28 days | 56 days | Soil | + + | + + |
| Stomatal conductance | Wheat | Alzahrani *et al*. (2018) | 20 | 4 mM K silicate | 50% FC | 45 days | 60 days | Soil | + + + | + + + |
|  | *Zygophyllum xanthoxylum* | Kang *et al*. (2016) | 12 | 2.5  mM K silicate | 30% FC | 10 days | 5 weeks | Vermiculture | + + + + | + + + |
|  | Cucumber | Ouzounidou *et al*. (2016) | 12 | 2.25 mM K silicate (foliar spray every 10 days) | Irrigation reduced by 50% | ~3 months | When fruits ripe | Soil | Stress-sensitive cultivar: + + +  Stress tolerant cultivar: + + + + | Both cultivars: + + + + |
|  | Strawberry | Moradtalab *et al*. (2019) | 2 | 0.6 mM Na silicate | 35% WHC | 4 weeks | 6 weeks | Hydroponics | + + + | + + + |
|  | Sunflower | Seleiman *et al*. (2019) | 0 | 10 t ha^-1^ biochar applied before sowing or 150 g ha^-1^ K silicate 30 and 55 DAS | 90% water deficit | From sowing until flowering | Flowering | Soil | Unknown | Biochar: + +  Silicate: + |
| Photosynthesis | Tomato | Shi *et al*. (2016) | 36 | 2.5 mM K silicate | 10% PEG-6000 | 7 days | 7 days after 5-leaf stage | Hydroponics | + + + + | + + + |
|  | Tomato | Cao *et al*. (2015) | 21 | 0.6 mM Na silicate | 1 % PEG-6000 | 12 days | 12 days after 9-leaf stage | Hydroponics | + + + | + + + |
|  | Wheat | Alzahrani *et al*. (2018) | 20 | 4 mM K silicate | 50% FC | 45 days | 60 days | Soil | + + + | + + + |
|  | *Zygophyllum xanthoxylum* | Kang *et al*. (2016) | 12 | 2.5  mM K silicate | 30% FC | 10 days | 5 weeks | Vermiculture | + + + + | + + + |
|  | Cucumber | Ouzounidou *et al*. (2016) | 12 | 2.25 mM K silicate (foliar spray every 10 d) | Irrigation reduced by 50% | ~3 months | When fruits ripe | Soil | Sensitive: + + +  Tolerant: + + + + | Sensitive: + + + +  Tolerant: + + + |
|  | Maize | Amin *et al*. (2018) | 7 | 100 mg kg^-1^ Ca silicate | 60 % FC | 3 weeks after germination until maturity | Maturity | Soil | Both cultivars: + + + | 45 and 70 days: + + + |
|  | Strawberry | Dehghanipoodeh *et al*. (2018) | 4 | 10 mM K silicate | 0.75 soil moisture depletion | 3 months | 7 months | 1:1:1 Soil:Peat:Perlite | + + + | + + + + |
|  | Strawberry | Moradtalab *et al*. (2019) | 2 | 0.6 mM Na silicate | 35% WHC | 4 weeks | 6 weeks | Hydroponics | + + + | + + + |
|  | Rice | Yang *et al*. (2019) | 1 | 4000 mg kg^-1^ Ca silicate slag | 50% FC | 28 days | 56 days | Soil | + + | + + + |
| Chlorophyll content | Wheat | Ma *et al*. (2016) | 36 | 204 mg kg^-1^ Na silicate | 55% RWC | 10 and 20 days | 10 and 20 days after heading | Soil | Unknown | 10 d: + + +  20 d: + + + |
|  | Tomato | Cao *et al*. (2015) | 21 | 0.6 mM Na silicate | 1 % PEG-6000 | 12 days | 12 days after 9-leaf stage | Hydroponics | + + + | + + + |
|  | Durum wheat | Meunier *et al*. (2017) | 17 | 1.5 mM K silicate | 12 % PEG | 30 days | 35 days | Hydroponics | + + + + | + + + + |
|  | Rapeseed | Hasanuzzaman *et al*. (2018) | 16 | 1 mM SiO_2_ | 20 % PEG | 48 hours | 14 days | Hydroponics | Unknown | + + |
|  | Mango | Helaly *et al*. (2017) | 14 | 1.5 mM K silicate | Water potential of -0.77 bars | 3 years | 23 years | Soil | All cultivars: + + + + + | All cultivars: + + + + |
|  | Barley | Hosseini *et al*. (2017) | 13 | 1 mM Na silicate | 190 g L^-1^ PEG-6000 | 5 days | 26 days | Hydroponics | N.s | N.s. |
|  | Cowpea | Merwad *et al*. (2018) | 13 | 2 mM K silicate (foliar spray 15, 30, 45 DAS) | 20 % WHC | 55 and 80 days | 80 days | Soil | + + | + + |
|  | *Zygophyllum xanthoxylum* | Kang *et al*. (2016) | 12 | 2.5  mM K silicate | 30% FC | 10 days | ~5 weeks | Vermiculture | + + + + | + + + + |
|  | Cucumber | Ouzounidou *et al*. (2016) | 12 | 2.25 mM K silicate (foliar spray every 10 days) | Irrigation reduced by 50% | 3 months | When fruits ripe | Soil | Sensitive: + + +  Tolerant: + + + + | Sensitive: + + +  Tolerant: + + + + |
|  | Wheat | Maghsoudi *et al*. (2015) | 11 | 6 mM Na silicate (foliar spray 30 DAS) | 40% FC | 20 days | 45 days | Soil | Tolerant: + + + +  Sensitive: + + +  (2 cultivars of each) | Tolerant: + + +  Sensitive: + + |
|  | Barley | Maillard *et al*. (2018) | 8 | 1.5 mM silicic acid | 190 g L^-1^ PEG-6000 | 4 days | 2 weeks | Hydroponics | No effect | N.s. |
|  | Tomato | Zhang *et al*. (2018) | 5 | 2.5 mM K silicate | 10% PEG-6000 | 7 days | 5-6 leaf stage | Hydroponics | + + + + | + + + |
|  | Tomato | Ali *et al*. (2018) | 4 | 1.5 mM silicic acid | 1% PEG-6000 | 3 weeks | 4 weeks | Hydroponics | Sensitive cultivar: + + +  Tolerant cultivar: n.s. | Sensitive cultivar: + + + + +  Tolerant cultivar: n.s. |
|  | Strawberry | Dehghanipoodeh *et al*. (2018) | 4 | 10 mM K silicate | 0.75 soil moisture depletion | 3 months | 7 months | 1:1:1 Soil:Peat:Perlite | + + + | N.s. |
|  | Rice | Wang *et al*. (2019) | 1 | 0.5 mM Na meta-silicate | 125 g L^-1^ PEG-6000 | 15 days | 5 weeks | Hydroponics | Unknown | + + + |
|  | Sugarcane | de Camargo *et al*. (2019) | 0 | 600 kg ha^-1^ Ca Mg silicate | 55% FC | 28 d | 6 months | Soil | + + + + | + + + + |
|  | Maize | Parveen *et al*. (2019) | 0 | 6 mM Na silicate | 60% FC | 3 weeks | 1 month | Soil | Both cultivars: + + + + + | Both cultivars: + + + + |
| Carotenoids | Cowpea | Merwad *et al*. (2018) | 13 | 2 mM K silicate (foliar spray 15, 30, 45 DAS) | 20 % WHC | 55 and 80 days | 80 days | Soil | + + | + + + |
|  | Tomato | Zhang *et al*. (2018) | 5 | 2.5 mM K silicate | 10% PEG-6000 | 7 days | 5-6 leaf stage | Hydroponics | + + + + | + + + |
|  | Maize | Parveen *et al*. (2019) | 0 | 6 mM Na silicate | 60% FC | 3 weeks | 1 month | Soil | Both cultivars: + + + + + | Both cultivars: n.s. |

*Search criteria:

- Search was carried out using Web of Science ([www.webofknowledge.com](http://www.webofknowledge.com)) database between 17/12/19 and 23/12/19 using the following search terms: Silicon AND drought; Silicon AND “osmotic stress”; Silicon AND salinity; Silicon AND “salt stress”, covering the last 5 years (01/01/2015 to 31/12/2019).
- Citation scores are based on numbers retrieved from web of science on 10/01/20
- Research articles only were included
- All papers from 2019 and 2018 were included, earlier publications were only included if cited >10-fold

**Effect size guide:

- Very large: under stress, levels with Si surpass those for control conditions **(+++++/-----)** with ‘+’ and ‘-‘ denoting positive and negative change respectively
- Large: under stress, levels with Si are similar to those for control conditions **(++++/----)**
- Medium: under stress, levels with Si are ~50% of those for control conditions **(+++/ ---)**
- Small: under stress, levels with Si are ~25% of those for control conditions **(++/--)**
- Very small: under stress, levels with Si are less than 25% of those for control conditions **(+/-)**
- No significant effect **(n.s.)**
