# Supplementary material for: Is Silicon a Panacea for Alleviating Drought and Salt Stress in Crops?
Source: Front Plant Sci. 2020 Aug 18;11:1221. doi: 10.3389/fpls.2020.01221 (PMC7461962; doi:10.3389/fpls.2020.01221)
Supplement: Supplementary file 2 [file Table_2.docx]

**Table 2: The impact of Silicon on plant Salinity Stress**

A literature search was carried out to evaluate how Si affects plant drought stress (see * for used criteria). The observed impacts of various parameters were classified from ‘non-significant’ to ‘very large’ (see **). Where multiple Si or stress treatments were used, data are reported for the most beneficial dose of Si and for the highest stress level only. The term ‘hydroponics’ was used to describe any study where the main source of nutrients was from a nutrient solution (e.g. includes studies using sand/perlite).

Abbreviations: relative water content (RWC); water use efficiency (WUE); days after sowing (DAS); field capacity (FC)

| **Measured variable** | **Species** | **Reference** | **No. citations** | **Level of Si** | **Level of stress** | **Length of stress treatment** | **Harvest growth stage** | **Growth conditions** | **Effects on growth (under stress)** | **Strength of Si effect** |
| --- | --- | --- | --- | --- | --- | --- | --- | --- | --- | --- |
| Oxidative damage | Sorghum | Liu *et al*. (2015) | 52 | 1.67 mM Na silicate (added 3 days before stress) | 100 mM NaCl | 2 hours | 12 days | Hydroponics | Unknown | – – – |
|  | Okra | Abbas *et al*. (2015) | 39 | 150 mg L^-1^ silicic acid (foliar spray 1 week after stress) | 6 dS m-1 | 2 weeks | 6 weeks | Hydroponics | Both cultivars: + + + + | Tolerant: – – –  Sensitive: – – – – |
|  | Tomato | Li *et al*. (2015) | 25 | 2 mM Na silicate | 150 mM Na | 15 days | 15 days after 6-leaf stage | Hydroponics | + + + | – – – |
|  | Tomato | Muneer and Jeong (2015) | 25 | 2.5 mM Na silicate | 50 mM NaCl | 5 days | 1 month | Hydroponics | Unknown | – – – – |
|  | Wheat | Alzahrani *et al*. (2018) | 20 | 4 mM K silicate | 200 mM NaCl | 45 days | 60 days | Soil | + + + | – – – |
|  | Pepper | Manivannan *et al*. (2016) | 19 | 1.8 mM K silicate | 50 mM NaCl | 15 days | 3 weeks | Hydroponics | + + + | – – – |
|  | Cucumber | Zhu *et al*. (2016) | 17 | 0.3 mM Na meta-silicate | 75 mM NaCl | 5, 10, and 15 days | 12, 17, and 22 days after 2-leaf stage | Hydroponics | 15 d: both cultivars: + + + | 5 d: both cultivars: – –  10 d and 15 d: both cultivars: – – – |
|  | Rice | Abdel-Haliem *et al*. (2017) | 16 | 50 mL Na silicate prepared from rice straw | 800 mM NaCl | 9 days | 29 days | Soil | Unknown | – – – – |
|  | Chickpea | Garg and Bhandari, (2016a) | 15 | 4 mM K silicate | 100 mM NaCl | 80 days | 3 months | Soil | Tolerant: + + +  Sensitive: + + | Both cultivars: – – – |
|  | *Glycyrrhiza uralensis* | Li *et al*. (2016) | 15 | 2 mM K silicate | 50 mM NaCl | 10, 20, 30 days | 40, 50, 60 days | Hydroponics | 10 d: n.s.  20 d: + + + + +  30 d: + + + + | 10 d: n.s.  20 d: – – – –  30 d: – – – – |
|  | *Glycyrrhiza uralensis* | Zhang *et al*. (2017) | 13 | 1 mM K silicate | 100 mM NaCl | 10 days | 10 days | Petri dish | + + + + + | – – – |
|  | Rice | Farooq *et al*. (2015) | 12 | 150 mg kg^-1^ Na silicate | 10 dS m^-1^ | From 45 days old to booting stage | Booting stage | Soil | Tolerant: + + +  Sensitive: + + | Both cultivars: – – – – – |
|  | Cucumber | Ouzounidou *et al*. (2016) | 12 | 2.25 mM K silicate (foliar spray every 10 days) | 150 mM NaCl | 2-3 months | When fruits ripe | Soil | Both cultivars: + + + | Sensitive: – – –  Tolerant: – – – |
|  | *Glycyrrhiza uralensis* | Zhang *et al*. (2018) | 12 | 0.1 g kg^-1^ K silicate | 6 g kg^-1^ NaCl | 70 and 110 days | 1 year 70 or 110 days | Soil | Unknown | 70 d: – – – –  110 d: – – – – |
|  | Pea | Shahid *et al*. (2015) | 10 | 150 mg L^-1^ K silicate (foliar spray 5 and 10 days after Na stress) | 6 dS m^-1^ | 25 and 60 days | 90 days | Hydroponics | + + + | – – – |
|  | Borage | Torabi *et al*. (2015) | 10 | 1.5 mM Na silicate | 120 mM NaCl | 35 days | 52 days | Hydroponics | + + + | – – – |
|  | Cucumber | Yin *et al*. (2019) | 10 | 0.3 mM Na meta-silicate | 75 mM NaCl | 5, 10, 15 days | 12, 17, 22 days after 2-leaf stage | Hydroponics | 15 d: + + + | 10 d: – – – |
|  | Rose | Soundararajan *et al*. (2018) | 9 | 1.8 mM Na silicate | 50 mM NaCl | 15 days | Unknown (In vitro grown acclimatised) | Hydroponics | + + + + | – – – – |
|  | Rapeseed | Hasanuzzaman *et al*. (2018) | 4 | 1 mM SiO_2_ | 200 mM NaCl | 48 hours | 14 days | Petri dish | Unknown | – – – |
|  | Maize | Bosnic *et al*. (2018) | 3 | 1.5 mM silicic acid (7 days before stress) | 40 mM NaCl | 3 weeks | 5 weeks | Hydroponics | + + + | – – – |
|  | Sunflower | Conceição *et al*. (2019) | 3 | 2 mM Na silicate | 150 mM NaCl | 2 weeks | 6 weeks | Hydroponics | Unknown | – – |
|  | *Phaseolus vulgaris* | Rady *et al*. (2019) | 3 | 6 mM K meta-silicate (foliar spray, 25, 35, 45 DAS) | 150 mM NaCl | 30 days | 55 days | Hydroponics | + + + | – – – |
|  | Wheat | Sienkiewicz-Cholewa *et al*. (2018) | 3 | Exp 1: 1 mM silicic acid  Exp 2: 1.5 mM | Exp 1: 70 mM NaCl  Exp 2: 100 mM | 10 days | 12 days | Hydroponics | Exp 1: n.s.  Exp 2: + | Both exps: – – – – |
|  | Wheat | Daoud *et al*. (2018) | 2 | 0.78 mM Na meta-silicate | 120 mM NaCl | 10, 21, 68, 108 days | 22, 43, 80, 120 days | Hydroponics | All growth stages: + + + | 22 d: + + + + +  Other growth stages: – – |
|  | *Glycyrrhiza uralensis* | Zhang *et al*. (2018b) | 2 | 0.6 g kg^-1^ SiO_2_ | 9 g kg^-1^ NaCl | 90, 120, 150 days | 1 year old and 90, 120, 150 days | Soil | 90 d: + + + +  120 d: n.s.  150 d: + + + + | 90 d: – –  120 d: – –  150 d: – – |
|  | Pepper | Akhoundnejad *et al*. (2018) | 1 | 2 mM K silicate | 150 mM NaCl | 30 days | 60 days | Hydroponics | Both cultivars: + + | Both cultivars: – – |
|  | *Acacia gerrardii* Benth | Al-Huqail *et al*. (2019) | 1 | 2 mM K silicate | 200 mM NaCl | 8 weeks | 3 months | Hydroponics | + + + | – – – – – |
|  | *Puccinellia distans* | Soleimannejad *et al*. (2019) | 1 | 1.5 mM Na silicate | 200 mM NaCl | 6 weeks | 7 weeks after 3-leaf stage | Hydroponics | + + + | – – |
|  | Canola | El-Shazoly (2019) | 0 | 1 mM Na silicate | 90 mM NaCl | 8 days | 8 days | Petri dish | + + + + + | N.s. |
|  | Rice | Lekklar *et al*. (2019) | 0 | 0.5 mM silicic acid | 60 mM NaCl | 7 and 14 days | 4 and 5 weeks | Hydroponics | 7 d: + + + +  14 d: + + + | Leaves: both: – – –  Roots: 7 d: + + + +  14 d: + + + |
| Anti-oxidative enzyme activity | Sorghum | Liu *et al*. (2015) | 52 | 1.67 mM Na silicate (added 3 days before stress) | 100 mM NaCl | 2 hours | 12 days | Hydroponics | Unknown | + |
|  | Okra | Abbas *et al*. (2015) | 39 | 150 mg L^-1^ silicic acid (foliar spray 1 week after stress) | Electrical conductivity of 6 dS m-1 | 2 weeks | 6 weeks | Hydroponics | Both cultivars: + + + + | Both cultivars: + + + + + |
|  | Tomato | Li *et al*. (2015) | 25 | 2 mM Na silicate | 150 mM Na | 15 days | 15 days after 6-leaf stage | Hydroponics | + + + | + + + |
|  | Tomato | Muneer and Jeong (2015) | 25 | 2.5 mM Na silicate | 50 mM NaCl | 5 days | 1 month | Hydroponics | Unknown | – – – – – |
|  | Wheat | Alzahrani *et al*. (2018) | 20 | 4 mM K silicate | 200 mM NaCl | 45 days | 60 days | Soil | + + + | + + + + + |
|  | Pepper | Manivannan *et al*. (2016) | 19 | 1.8 mM K silicate | 50 mM NaCl | 15 days | 3 weeks | Hydroponics | + + + | + + + + + |
|  | Carnation | Soundararajan *et al*. (2015) | 18 | 50 mg K silicate | 100 mM NaCl | 30 days | 30 days | Agar | + + + + | General – – |
|  | Rice | Abdel-Haliem *et al*. (2017) | 16 | 50 mL Na silicate prepared from rice straw | 800 mM NaCl | 9 days | 29 days | Soil | Unknown | + + + + + |
|  | Chickpea | Garg and Bhandari, (2016a) | 15 | 4 mM K silicate | 100 mM NaCl | 80 days | 3 months | Soil | Tolerant: + + +  Sensitive: + + | Both cultivars: + + + + + |
|  | *Glycyrrhiza uralensis* | Li *et al*. (2016) | 15 | 2 mM K silicate | 50 mM NaCl | 10, 20, 30 days | 40, 50, 60 days | Hydroponics | 10 d: n.s.  20 d: + + + + +  30 d: + + + + | 10 d: + + +  20 d: + + + + +  30 d: + + + + + |
|  | *Lonicera japonica* | Gengmao *et al*. (2015) | 13 | 0.5 g L^-1^ K silicate | 200 mM NaCl | 30 and 40 days | 70 days | Hydroponics | + + + | – – |
|  | *Glycyrrhiza uralensis* | Zhang *et al*. (2017) | 13 | 1 mM K silicate | 100 mM NaCl | 10 days | 10 days | Petri dish | + + + + + | N.s. |
|  | Rice | Farooq *et al*. (2015) | 12 | 150 mg kg^-1^ Na silicate | 10 dS m^-1^ | From 45 days old to booting stage | Booting stage | Soil | Tolerant: + + +  Sensitive: + + | Both cultivars: + + + + + |
|  | *Glycyrrhiza uralensis* | Zhang *et al*. (2018) | 12 | 0.1 g kg^-1^ K silicate | 6 g kg^-1^ NaCl | 70 and 110 days | 1 year 70 or 110 days | Soil | Unknown | 70 d: n.s.  110 d: + + |
|  | Pea | Shahid *et al*. (2015) | 10 | 150 mg L^-1^ K silicate (foliar spray 5 and 10 days after stress) | 6 dS m^-1^ | 25 and 60 days | 90 days | Hydroponics | + + + | Generally + + + + + |
|  | Borage | Torabi *et al*. (2015) | 10 | 1.5 mM Na silicate | 120 mM NaCl | 35 d | 52 days | Hydroponics | + + + | Generally + + + + + |
|  | Cucumber | Yin *et al*. (2019) | 10 | 0.3 mM Na meta-silciate | 75 mM NaCl | 5, 10, 15 days | 12, 17, 22 days after 2-leaf stage | Hydroponics | 15 d: + + + | 10 d: general – – – |
|  | Rose | Soundararajan *et al*. (2018) | 9 | 1.8 mM Na silicate | 50 mM NaCl | 15 days | Unknown (In vitro grown acclimatised) | Hydroponics | + + + + | + + + + |
|  | Rice | Farooq *et al*. (2019) | 7 | 150 mg kg^-1^ Na silicate | 10 dS m^-1^ | Whole experiment | Booting stage | Soil | + + | + + + + + |
|  | Rice | Das *et al*. (2018) | 5 | 2 mM Na meta-silicate | 100 mM NaCl | 21 days | 23 days | Petri dish | Unknown | Both cultivars: + |
|  | Rapeseed | Hasanuzzaman *et al*. (2018) | 4 | 1 mM SiO_2_ | 200 mM NaCl | 48 hours | 14 days | Petri dish | Unknown | + + + + |
|  | Sunflower | Conceição *et al*. (2019) | 3 | 2 mM Na silicate | 150 mM NaCl | 2 weeks | 6 weeks | Hydroponics | Unknown | General + + + + |
|  | *Phaseolus vulgaris* | Rady *et al*. (2019) | 3 | 6 mM K meta-silicate (foliar spray, 25, 35, 45 DAS) | 150 mM NaCl | 30 days | 55 days | Hydroponics | + + + | + + + + + |
|  | *Brassica juncea* | Siddiqui *et al*. (2018) | 3 | 0.8 mM Na silicate (foliar spray 20 DAS) | 150 mM NaCl | 30 days | 45 days | Soil | + + + + | General + + + + + |
|  | Wheat | Daoud *et al*. (2018) | 2 | 0.78 mM Na meta-silicate | 120 mM NaCl | 10, 21, 68, 108 days | 22, 43, 80 and 120 days | Hydroponics | All growth stages: + + + | All growth stages: + + + + + |
|  | *Glycyrrhiza uralensis* | Zhang *et al*. (2018b) | 2 | 0.6 g kg^-1^ SiO_2_ | 9 g kg^-1^ NaCl | 90, 120, 150 days | 1 year old and 90, 120, 150 days | Soil | 90 d: + + + +  120 d: n.s.  150 d: + + + + | 90 d: + + + +  120 d: + + + + +  150 d: general + + + |
|  | Coriander | Al-Garni *et al*. (2019) | 1 | 150 mL 0.1% w/v K silicate as foliar spray 1 week after stress treatment | 75 mM NaCl | 3 weeks | 5 weeks | Soil | + + + | + + + + + |
|  | *Acacia gerrardii* Benth | Al-Huqail *et al*. (2019) | 1 | 2 mM K silicate | 200 mM NaCl | 8 weeks | 3 months | Hydroponics | + + + | + + + + + |
|  | Maize | Khan *et al*. (2018) | 1 | 2 mM silicic acid (5 days before stress) | 60 mM NaCl | 40 days | 50 days | Hydroponics | Tolerant: +  Sensitive: + + + | Both cultivars: general + + + + |
|  | Maize | Delavar *et al*. (2018) | 0 | 2 mM Na meta-silicate (2 weeks before stress) | 150 mM NaCl | 2 weeks | 5 weeks | Hydroponics | + + + | General – – – |
|  | Canola | El-Shazoly (2019) | 0 | 1 mM Na silicate | 90 mM NaCl | 8 days | 8 days | Petri dish | + + + + + | General – – |
|  | Maize | Fialová *et al*. (2018) | 0 | 2.5 mM Na silicate | 150 NaCl | 10 days | 13 days | Hydroponics | Unknown | Sensitive: + + + + +  Tolerant: general – – |
|  | Rice | Lekklar *et al*. (2019) | 0 | 0.5 mM silicic acid | 60 mM NaCl | 7 and 14 days | 4 and 5 weeks | Hydroponics | 7 d: + + + +  14 d: + + + | Both times: + + + + + |
| Anti-oxidant content | Wheat | Alzahrani *et al*. (2018) | 20 | 4 mM K silicate | 200 mM NaCl | 45 days | 60 days | Soil | + + + | + + + + + |
|  | Chickpea | Garg and Bhandari, (2016a) | 15 | 4 mM K silicate | 100 mM NaCl | 80 days | 3 months | Soil | Tolerant: + + +  Sensitive: + + | Tolerant: + + +  Sensitive: + + |
|  | Cucumber | Ouzounidou *et al*. (2016) | 12 | 2.25 mM K silicate (foliar spray every 10 days) | 150 mM NaCl | 2-3 months | When fruits ripe | Soil | Both cultivars: + + + | Sensitive: + + +  Tolerant: + + |
|  | *Glycyrrhiza uralensis* | Zhang *et al*. (2018) | 12 | 0.1 g kg^-1^ K silicate | 6 g/kg NaCl | 70 and 110 days | 1 year 70 or 110 days | Soil | Unknown | 70 d: + +  110 d: + + + + + |
|  | Rose | Soundararajan *et al*. (2018) | 9 | 1.8 mM Na silicate | 50 mM NaCl | 15 days | Unknown (In vitro grown acclimatised) | Hydroponics | + + + + | + + + |
|  | Rice | Das *et al*. (2018) | 5 | 2 mM Na meta-silicate | 100 mM NaCl | 21 days | 23 days | Petri dish | Unknown | Both cultivars: + |
|  | Rapeseed | Hasanuzzaman *et al*. (2018) | 4 | 1 mM SiO_2_ | 200 mM NaCl | 48 hours | 14 days | Petri dish | Unknown | + + + |
|  | *Acacia gerrardii* Benth | Al-Huqail *et al*. (2019) | 1 | 2 mM K silicate | 200 mM NaCl | 8 weeks | 3 months | Hydroponics | + + + | + + + |
|  | Canola | El-Shazoly (2019) | 0 | 1 mM Na silicate | 90 mM NaCl | 8 days | 8 days | Petri dish | + + + + + | N.s. |
|  | Strawberry | Yaghubi *et al*. (2019) | 0 | 1500 ppm K silicate | 50 mM NaCl | 60 days | 3 months | Hydroponics | Both cultivars: + + + | One cultivar: general + +  Other: n.s. |
| Compatible solutes | Sorghum | Yin *et al*. (2016) | 42 | 0.83 mM silicic acid | 100 mM NaCl | 1, 3, and 7 days | 16, 30, and 24 days | Hydroponics | 1 d and 3 d: n.s.  7 d: + + + + | 1 d and 3 d: general + + + +  7 d: + + + + + |
|  | Okra | Abbas *et al*. (2015) | 39 | 150 mg L^-1^ silicic acid (foliar spray 1 week after stress) | 6 dS m^-1^ | 2 weeks | 6 weeks | Hydroponics | Both cultivars: + + + + | + + + + + |
|  | Cucumber | Wang *et al*. (2015) | 33 | 0.8 mM silicic acid 6 days before stress | 65 mM NaCl | 1.5, 3.5, 7.5 days | 17.5, 19.5, 23.5 days | Hydroponics | + + after 7.5 days stress | 1.5 d: + + + +  3.5 d: n.s.  7.5 d: + + |
|  | Wheat | Alzahrani *et al*. (2018) | 20 | 4 mM K silicate | 200 mM NaCl | 45 days | 60 days | Soil | + + + | + + + + + |
|  | Cucumber | Zhu *et al*. (2016) | 17 | 0.3 mM Na meta-silicate | 75 mM NaCl | 5, 10, and 15 days | 12, 17, and 22 days after 2-leaf stage | Hydroponics | 15 d: both cultivars: + + + | Leaf: 5 d: sensitive: n.s.; tolerant: – –  10 d: both cultivars: – –  15 d: sensitive: – –; tolerant: – – –  Roots: sensitive: all times, + + + + +; tolerant: same pattern as in leaves |
|  | Rice | Abdel-Haliem *et al*. (2017) | 16 | 50 mL Na silicate prepared from rice straw | 800 mM NaCl | 9 days | 29 days | Soil | Unknown | – – – – |
|  | *Glycyrrhiza uralensis* | Zhang *et al*. (2017) | 13 | 1 mM K silicate | 100 mM NaCl | 10 days | 10 days | Petri dish | + + + + + | + + + + + |
|  | Cucumber | Ouzounidou *et al*. (2016) | 12 | 2.25 mM K silicate (foliar spray every 10 days) | 150 mM NaCl | 2-3 months | When fruits ripe | Soil | Both cultivars: + + + | Sensitive: – – – –  Tolerant: – – – |
|  | *Glycyrrhiza uralensis* | Zhang *et al*. (2018) | 12 | 0.1 g kg^-1^ K silicate | 6 g kg^-1^ NaCl | 70 and 110 days | 1 year 70 or 110 days | Soil | Unknown | 70 d: n.s.  110 d: + + |
|  | Pea | Shahid *et al*. (2015) | 10 | 150 mg L^-1^ K silicate (foliar spray 5 and 10 days after stress) | 6 dS m^-1^ | 25 and 60 days | 90 days | Hydroponics | + + + | + + + + + |
|  | Borage | Torabi *et al*. (2015) | 10 | 1.5 mM Na silicate | 120 mM NaCl | 35 d | 52 d | Hydroponics | + + + | + + + + + |
|  | Cucumber | Yin *et al*. (2019) | 10 | 0.3 mM Na meta-silciate | 75 mM NaCl | 5, 10, 15 days | 12, 17, 22 days after 2-leaf stage | Hydroponics | 15 d: + + + | All times, roots and leaves: + + + + + |
|  | Sunflower | Conceição *et al*. (2019) | 3 | 2 mM Na silicate | 150 mM NaCl | 2 weeks | 6 weeks | Hydroponics | Unknown | + + + + + |
|  | *Phaseolus vulgaris* | Rady *et al*. (2019) | 3 | 6 mM K meta-silicate (foliar spray, 25, 35, 45 DAS) | 150 mM NaCl | 30 days | 55 days | Hydroponics | + + + | + + + + + |
|  | *Brassica juncea* | Siddiqui *et al*. (2018) | 3 | 0.8 mM Na silicate (foliar spray 20 DAS) | 150 mM NaCl | 30 days | 45 days old | Soil | + + + + | N.s. |
|  | Wheat | Sienkiewicz-Cholewa *et al*. (2018) | 3 | Exp 1: 1 mM silicic acid  Exp 2: 1.5 mM | Exp 1: 70 mM NaCl  Exp 2: 100 mM | 10 d | 12 d | Hydroponics | Exp 1: n.s.  Exp 2: + | Both exps: general – – |
|  | *Glycyrrhiza uralensis* | Zhang *et al*. (2018b) | 2 | 0.6 g kg^-1^ SiO_2_ | 9 g kg^-1^ NaCl | 90, 120, 150 days | 1 year old and 90, 120, 150 days | Soil | 90 d: + + + +  120 d: n.s.  150 d: + + + + | 90 d: general + +  120 d: + +  150 d: n.s. |
|  | *Acacia gerrardii* Benth | Al-Huqail *et al*. (2019) | 1 | 2 mM K silicate | 200 mM NaCl | 8 weeks | 3 months | Hydroponics | + + + | + + + + + |
|  | *Puccinellia distans* | Soleimannejad *et al*. (2019) | 1 | 1.5 mM Na silicate | 200 mM NaCl | 6 weeks | 7 weeks after 3-leaf stage | Hydroponics | + + + | General – – |
|  | Maize | Delavar *et al*. (2018) | 0 | 2 mM Na meta-silicate (2 weeks before stress) | 150 mM NaCl | 2 weeks | 5 weeks | Hydroponics | + + + | General – – in shoots and + + in roots |
| Nutrient content | Chickpea | Garg and Bhandari, (2016) | 38 | 4 mM K silicate | 100 mM NaCl | 9 weeks | 80 days | Soil | Both cultivars: + + | Both cultivars: + + |
|  | *Aloe vera* | Xu *et al*. (2015) | 27 | 2 mM K silicate | 100 mM NaCl | 30 and 120 days | 30 and 120 days after 7-leaf stage | Hydroponics | + + + + | 30 d: general + + |
|  | Tomato | Li *et al*. (2015) | 25 | 2 mM Na silicate | 150 mM Na | 15 days | 15 days after 6-leaf stage | Hydroponics | + + + | + + + |
|  | Wheat | Alzahrani *et al*. (2018) | 20 | 4 mM K silicate | 200 mM NaCl | 45 days | 60 days | Soil | + + + | + + + |
|  | Rice | Abdel-Haliem *et al*. (2017) | 16 | 50 mL Na silicate prepared from rice straw | 800 mM NaCl | 9 days | 29 days | Soil | Unknown | + + + + |
|  | Chicory | D’Imperio *et al*. (2018) | 9 | 3.6 mM K meta-silicate | 50 mM NaCl | 6 weeks | 2 months | Hydroponics | N.s. | Edible parts: n.s. |
|  | *Phaseolus vulgaris* | Rady *et al*. (2019) | 3 | 6 mM K meta-silicate (foliar spray, 25, 35, 45 DAS) | 150 mM NaCl | 30 days | 55 days | Hydroponics | + + + | + + + |
|  | Sunflower | Calero Hurtado *et al*. (2019) | 0 | 28.6 mM foliar spray at V4-V8 (applied once per week, 3 times total) or 2 mM Si in nutrient solution or both (stabilised Na and K silicate) | 100 mM NaCl | 1 month | 7 weeks | Hydroponics | Foliar: + +  Solution: + +  Both: + + + | Foliar and solution: in general, n.s.  Both: general + |
|  | Sorghum | Calero Hurtado *et al*. (2019) | 0 | 28.6 mM foliar spray at S2-S4 (applied once per week, 3 times total) or 2 mM Si in nutrient solution or both (stabilised Na and K silicate) | 100 mM NaCl | 1 month | 7 weeks | Hydroponics | Foliar: no effect  Solution: + +  Both: + + | Foliar: general n.s.  Solution and both: general + |
|  | Tomato | Costan *et al*. (2019) | 0 | 2 mM K silicate | 50 mM NaCl | 6 months | 6 months after 2-leaf stage | Hydroponics | N.s. | N.s. |
|  | Maize | Delavar *et al*. (2018) | 0 | 2 mM Na meta-silicate (2 weeks before stress) | 150 mM NaCl | 2 weeks | 5 weeks | Hydroponics | + + + | – – – – |
| Na concentration | Sorghum | Liu *et al*. (2015) | 52 | 1.67 mM Na silicate (3 days before stress) | 100 mM NaCl | 2 hours | 12 days | Hydroponics | Unknown | Shoot and root: n.s. |
|  | Sorghum | Yin *et al*. (2016) | 42 | 0.83 mM silicic acid | 100 mM NaCl | 1, 3, and 7 days | 16, 30, and 24 days | Hydroponics | 1 d and 3 d: n.s.  7 d: + + + + | 1 d: leaf/stem: – –, root: + +  3 d: leaf: – – –, stem: – –, root: +  7 d: leaf: – – –, stem/root: – – |
|  | Okra | Abbas *et al*. (2015) | 39 | 150 mg L^-1^ silicic acid (foliar spray 1 week after stress) | 6 dS m^-1^ | 2 weeks | 6 weeks | Hydroponics | Both cultivars: + + + + | Shoots: Tolerant: – – – – Sensitive: – –  Roots: Tolerant: – – – Sensitive: – – – – – |
|  | Chickpea | Garg and Bhandari, (2016) | 38 | 4 mM K silicate | 100 mM NaCl | 9 weeks | 80 days | Soil | Both cultivars: + + | Tolerant cultivar: – – –  Sensitive cultivar: – – |
|  | Cucumber | Wang *et al*. (2015) | 33 | 0.8 mM silicic acid 6 days before stress | 65 mM NaCl | 1.5, 3.5, 7.5 days | 17.5, 19.5, 23.5 days | Hydroponics | + + after 7.5 days stress | 1.5 d: leaf/stem: n.s.; root: +  3.5 d: leaf: – ; stem/root: n.s.  7.5 d: leaf: – –; stem/root n.s. |
|  | Cucumber | Zhu *et al*. (2015) | 28 | 0.3 mM Na silicate | 75 mM NaCl | 15 days | 1 month | Hydroponics | Sensitive: + + +  Tolerant: + + + + | Shoots and roots: both cultivars: – – |
|  | *Aloe vera* | Xu *et al*. (2015) | 27 | 2 mM K silicate | 100 mM NaCl | 30 and 120 days | 30 and 120 days after 7-leaf stage | Hydroponics | + + + + | 30 d: root/stem/leaf: – – |
|  | Tomato | Li *et al*. (2015) | 25 | 2 mM Na silicate | 150 mM Na | 15 days | 15 days after 6-leaf stage | Hydroponics | + + + | Leaf, stem, root: – – – |
|  | Wheat | Alzahrani *et al*. (2018) | 20 | 4 mM K silicate | 200 mM NaCl | 45 days | 60 days | Soil | + + + | – – – |
|  | Chickpea | Garg and Bhandari, (2016a) | 15 | 4 mM K silicate | 100 mM NaCl | 80 days | 3 months | Soil | Tolerant: + + +  Sensitive: + + | Both cultivars: – – – (leaves/roots) |
|  | *Glycyrrhiza uralensis* | Li *et al*. (2016) | 15 | 2 mM K silicate | 50 mM NaCl | 10, 20, 30 days | 40, 50, 60 days | Hydroponics | 10 d: n.s.  20 d: + + + + +  30 d: + + + + | Shoots: 10 d: – – –  20 d: n.s.  30 d: – – – – |
|  | Rice | Flam-Shepherd *et al*. (2018) | 14 | 1.67 mM Na silicate | 50 mM NaCl | 19 days | 21 days | Hydroponics | Both cultivars: + + + + | Shoot: both cultivars: – – –  Root: both cultivars: n.s. |
|  | Wheat | Azeem *et al*. (2015) | 12 | 40 mM Na silicate (8h seed priming or fertilisation) | 120 mM NaCl | 21 days | 21 days | Hydroponics | Seed priming: + + + +  Fertilisation: + + + | Seed priming: n.s.  Fertilisation: – – |
|  | Rice | Farooq *et al*. (2015) | 12 | 150 mg kg^-1^ Na silicate | 10 dS m^-1^ | From 45 days old to booting stage | Booting stage | Soil | Tolerant: + + +  Sensitive: + + | Shoot: both cultivars: – – – – – |
|  | Rice | Mahdieh *et al*. (2015) | 12 | 3 mM Na silicate | 100 mM NaCl | 4 days | 30 days | Hydroponics | Both cultivars: n.s. | Shoot: both cultivars: – – – |
|  | *Glycyrrhiza uralensis* | Zhang *et al*. (2018) | 12 | 0.1 g kg^-1^ K silicate | 6 g kg^-1^ NaCl | 70 and 110 days | 1 year 70 or 110 days | Soil | Unknown | 70 d: root/stem/leaf: n.s.  110 d: root/stem: – –, leaf: n.s. |
|  | Pea | Shahid *et al*. (2015) | 10 | 150 mg L^-1^ K silicate (foliar spray 5 and 10 days after stress) | 6 dS m^-1^ | 25 and 60 days | 90 days | Hydroponics | + + + | Root: – – –  Shoot: – – |
|  | Chicory | D’Imperio *et al*. (2018) | 9 | 3.6 mM K meta-silicate | 50 mM NaCl | 6 weeks | 2 months | Hydroponics | N.s. | Edible parts: – – |
|  | Rose | Soundararajan *et al*. (2018) | 9 | 1.8 mM Na silicate | 50 mM NaCl | 15 days | Unknown (In vitro grown acclimatised) | Hydroponics | + + + + | Root: – – – |
|  | Maize | Bosnic *et al*. (2018) | 3 | 1.5 mM silicic acid (7 days before stress) | 40 mM NaCl | 3 weeks | 5 weeks | Hydroponics | + + + | Roots: – –  Shoots: + + + + + |
|  | Wheat | Daoud *et al*. (2018) | 2 | 0.78 mM Na meta-silicate | 120 mM NaCl | 10, 21, 68, 108 days | 22, 43, 80 and 120 days | Hydroponics | All growth stages: + + + | Leaf: all growth stages: – – – |
|  | Rice | Das *et al*. (2019) | 2 | 2 mM Na meta-silicate | 100 mM NaCl | 21 days | 23 days | Hydroponics | Unknown | Sensitive cultivar: – –  Tolerant cultivar: – |
|  | *Acacia gerrardii* Benth | Al-Huqail *et al*. (2019) | 1 | 2 mM K silicate | 200 mM NaCl | 8 weeks | 3 months | Hydroponics | + + + | Root and shoots: – – – |
|  | Durum Wheat | Bijanzadeh and Egan, (2018) | 1 | 1.5 mM Na silicate (3 hour seed priming) | 200 mM NaCl | 7 days | 7 days | Petri dish | Tolerant: + + +  Sensitive: + + + + | Leaf: tolerant: – – – –  Sensitive: – – – |
|  | Date palm | Jana *et al*. (2019) | 1 | 5 mM Na silicate | 300 mM NaCl | 8 weeks | 120 days | Soil | N.s. | Leaves and roots: n.s. |
|  | *Puccinellia distans* | Soleimannejad *et al*. (2019) | 1 | 1.5 mM Na silicate | 200 mM NaCl | 6 weeks | 7 weeks after 3-leaf stage | Hydroponics | + + + | Shoot and root: – – – |
|  | Sorghum | Calero Hurtado *et al*. (2019) | 0 | 28.6 mM foliar spray at S2-S4 (applied once per week, 3 times total) or 2 mM Si in nutrient solution or both (stabilised Na and K silicate) | 100 mM NaCl | 1 month | 7 weeks | Hydroponics | Foliar: no effect  Solution: + +  Both: + + | Shoots and roots:  Foliar: – –  Solution: – – –  Both: – – – |
|  | Sunflower | Calero Hurtado *et al*. (2019) | 0 | 28.6 mM foliar spray at V4-V8 (applied once per week, 3 times total) or 2 mM Si in nutrient solution or both (stabilised Na and K silicate) | 100 mM NaCl | 1 month | 7 weeks | Hydroponics | Foliar: + +  Solution: + +  Both: + + + | Shoots and roots:  All Si applications: – – |
|  | Maize | Delavar *et al*. (2018) | 0 | 2 mM Na meta-silicate (2 weeks before stress) | 150 mM NaCl | 2 weeks | 5 weeks | Hydroponics | + + + | Roots and shoots: – – – – |
|  | Strawberry | Yaghubi *et al*. (2019) | 0 | 1500 ppm K silicate | 50 mM NaCl | 60 days | 3 months | Hydroponics | Both cultivars: + + + | Leaves: both cultivars: – – –  Roots: both cultivars: – – |
| Relative water content | Sorghum | Liu *et al*. (2015) | 52 | 1.67 mM Na silicate (added 3 days before stress) | 100 mM NaCl | 2 hours | 12 days | Hydroponics | Unknown | + + + |
|  | Okra | Abbas *et al*. (2015) | 39 | 150 mg L^-1^ silicic acid (foliar spray 1 week after stress) | 6 dS m^-1^ | 2 weeks | 6 weeks | Hydroponics | Both cultivars: + + + + | Tolerant: + + + +  Sensitive: + + + |
|  | Chickpea | Garg and Bhandari, (2016) | 38 | 4 mM K silicate | 100 mM NaCl | 9 weeks | 80 days | Soil | Both cultivars: + + | Both cultivars: + + |
|  | Cucumber | Zhu *et al*. (2015) | 28 | 0.3 mM Na silicate | 75 mM NaCl | 15 days | 1 month | Hydroponics | Sensitive: + + +  Tolerant: + + + + | Both cultivars: + + + |
|  | Tomato | Li *et al*. (2015) | 25 | 2 mM Na silicate | 150 mM Na | 15 days | 15 days after 6-leafstage | Hydroponics | + + + | + + + |
|  | Wheat | Alzahrani *et al*. (2018) | 20 | 4 mM K silicate | 200 mM NaCl | 45 days | 60 days | Soil | + + + | + + + |
|  | Rice | Farooq *et al*. (2015) | 12 | 150 mg kg^-1^ Na silicate | 10 dS m^-1^ | From 45 days old to booting stage | Booting stage | Soil | Tolerant: + + +  Sensitive: + + | Tolerant: + + + +  Sensitive: + + + |
|  | Chicory | D’Imperio *et al*. (2018) | 9 | 3.6 mM K meta-silicate | 50 mM NaCl | 6 weeks | 2 months | Hydroponics | N.s. | N.s. |
|  | *Phaseolus vulgaris* | Rady *et al*. (2019) | 3 | 6 mM K meta-silicate (foliar spray, 25, 35, 45 DAS) | 150 mM NaCl | 30 days | 55 days | Hydroponics | + + + | + + + |
|  | *Glycyrrhiza uralensis* | Zhang *et al*. (2018b) | 2 | 0.6 g kg^-1^ SiO_2_ | 9 g kg^-1^ NaCl | 90, 120, 150 days | 1 year old and 90, 120, 150 days | Soil | 90 d: + + + +  120 d: n.s.  150 d: + + + + | 90 d: + + +  120 d: + + +  150 d: + + + + |
|  | Coriander | Al-Garni *et al*. (2019) | 1 | 150 mL 0.1% w/v K silicate as foliar spray 1 week after stress treatment | 75 mM NaCl | 3 weeks | 5 weeks | Soil | + + + | + + + |
|  | *Puccinellia distans* | Soleimannejad *et al*. (2019) | 1 | 1.5 mM Na silicate | 200 mM NaCl | 6 weeks | 7 weeks after 3-leaf stage | Hydroponics | + + + | + + + + |
| Water use efficiency | Okra | Abbas *et al*. (2015) | 39 | 150 mg L^-1^ silicic acid (foliar spray 1 week after stress) | 6 dS m^-1^ | 2 weeks | 6 weeks | Hydroponics | Both cultivars: + + + + | Tolerant: + + + +  Sensitive: + + + + + |
|  | Wheat | Alzahrani *et al*. (2018) | 20 | 4 mM K silicate | 200 mM NaCl | 45 days | 60 days | Soil | + + + | N.s. |
|  | Cucumber | Ouzounidou *et al*. (2016) | 12 | 2.25 mM K silicate (foliar spray every 10 days) | 150 mM NaCl | 2-3 months | When fruits ripe | Soil | Both cultivars: + + + | Sensitive: + + +  Tolerant: + + + + |
|  | Pea | Shahid *et al*. (2015) | 10 | 150 mg L^-1^ K silicate (foliar spray 5 and 10 days after Na stress) | 6 dS m^-1^ | 25 days | 90 days | Hydroponics | + + + | + + + + |
|  | *Brassica juncea* | Siddiqui *et al*. (2018) | 3 | 0.8 mM Na silicate (foliar spray 20 DAS) | 150 mM NaCl | 30 days | 45 days old | Soil | + + + + | + + + + |
|  | *Acacia gerrardii* Benth | Al-Huqail *et al*. (2019) | 1 | 2 mM K silicate | 200 mM NaCl | 8 weeks | 3 months | Hydroponics | + + + | – – – |
|  | Maize | Khan *et al*. (2018) | 1 | 2 mM silicic acid (5 days before stress) | 60 mM NaCl | 40 days | 50 days | Hydroponics | Tolerant: +  Sensitive: + + + | Tolerant: + + + + +  Sensitive: n.s. |
| Transpiration | Sorghum | Liu *et al*. (2015) | 52 | 1.67 mM Na silicate (added 3 days before stress) | 100 mM NaCl | 2 hours | 12 days | Hydroponics | Unknown | + + |
|  | Okra | Abbas *et al*. (2015) | 39 | 150 mg L^-1^ silicic acid (foliar spray 1 week after stress) | Electrical conductivity of 6 dS m-1 | 2 weeks | 6 weeks | Hydroponics | Both cultivars: + + + + | Tolerant: + + + +  Sensitive: + + + |
|  | Cucumber | Wang *et al*. (2015) | 33 | 0.8 mM silicic acid 6 days before stress | 65 mM NaCl | 1.5, 3.5, 7.5 days | 17.5, 19.5, 23.5 days | Hydroponics | + + after 7.5 days stress | 1.5 d: n.s.  3.5 d: + + +  7.5 d: + + + |
|  | Cucumber | Zhu *et al*. (2015) | 28 | 0.3 mM Na silicate | 75 mM NaCl | 15 days | 1 month | Hydroponics | Sensitive: + + +  Tolerant: + + + + | Sensitive: + + +  Tolerant: + + + + |
|  | Tomato | Li *et al*. (2015) | 25 | 2 mM Na silicate | 150 mM Na | 15 days | 15 days after 6-leaf stage | Hydroponics | + + + | + + + |
|  | Wheat | Alzahrani *et al*. (2018) | 20 | 4 mM K silicate | 200 mM NaCl | 45 days | 60 days | Soil | + + + | + + + |
|  | Pepper | Manivannan *et al*. (2016) | 19 | 1.8 mM K silicate | 50 mM NaCl | 15 days | 3 weeks | Hydroponics | + + + | + + + + |
|  | *Lonicera japonica* | Gengmao *et al*. (2015) | 13 | 0.5 g L^-1^ K silicate | 200 mM NaCl | 30 and 40 days | 70 days | Hydroponics | + + + | – – – – – |
|  | Rice | Farooq *et al*. (2015) | 12 | 150 mg kg^-1^ Na silicate | 10 dS m^-1^ | From 45 days old to booting stage | Booting stage | Soil | Tolerant: + + +  Sensitive: + + | Tolerant: – – – – –  Sensitive: + + + |
|  | Cucumber | Ouzounidou *et al*. (2016) | 12 | 2.25 mM K silicate (foliar spray every 10 days) | 150 mM NaCl | 2-3 months | When fruits ripe | Soil | Both cultivars: + + + | Both cultivars: + + + + + |
|  | Pea | Shahid *et al*. (2015) | 10 | 150 mg L^-1^ K silicate (foliar spray 5 and 10 days after Na stress) | 6 dS m^-1^ | 25 days | 90 days | Hydroponics | + + + | + + + |
|  | Chicory | D’Imperio *et al*. (2018) | 9 | 3.6 mM K meta-silicate | 50 mM NaCl | 6 weeks | 2 months | Hydroponics | N.s. | N.s. |
|  | *Phaseolus vulgaris* | Rady *et al*. (2019) | 3 | 6 mM K meta-silicate (foliar spray, 25, 35, 45 DAS) | 150 mM NaCl | 30 days | 55 days | Hydroponics | + + + | + + + |
|  | Cucumber | Zhu *et al*. (2019) | 3 | 0.3 mM Si | 75 mM Na | 10 and 15 days | 15 days after 2-leaf stage | Hydroponics | + + + | + + + |
|  | Pepper | Akhoundnejad *et al*. (2018) | 1 | 2 mM K silicate | 150 mM NaCl | 30 days | 60 days | Hydroponics | Both cultivars: + + | Both cultivars: + + |
|  | *Acacia gerrardii* Benth | Al-Huqail *et al*. (2019) | 1 | 2 mM K silicate | 200 mM NaCl | 8 weeks | 3 months | Hydroponics | + + + | + + + |
|  | Date palm | Jana *et al*. (2019) | 1 | 5 mM Na silicate | 300 mM NaCl | 8 weeks | 120 days old | Soil | N.s. | N.s. |
|  | Maize | Khan *et al*. (2018) | 1 | 2 mM silicic acid (5 days before Na) | 60 mM NaCl | 40 days | 50 days | Hydroponics | Tolerant: +  Sensitive: + + + | Both cultivars: n.s. |
|  | *Puccinellia distans* | Soleimannejad *et al*. (2019) | 1 | 1.5 mM Na silicate | 200 mM NaCl | 6 weeks | 7 weeks after 3-leaf stage | Hydroponics | + + + | – – – – – |
|  | Potato | Kafi *et al*. (2019) | 0 | 1000 ppm SiO_2_ (foliar spray 40 and 50 days after planting) | 12 dS m^-1^ | 27 days | 57 days and 5 months | Soil | + | – – – – – |
| Stomatal conductance | Sorghum | Liu *et al*. (2015) | 52 | 1.67 mM Na silicate (added 3 days before stress) | 100 mM NaCl | 2 hours | 12 days | Hydroponics | Unknown | + + + |
|  | Okra | Abbas *et al*. (2015) | 39 | 150 mg L^-1^ silicic acid (foliar spray 1 week after stress) | 6 dS m^-1^ | 2 weeks | 6 weeks | Hydroponics | Both cultivars: + + + + | Tolerant: + + + + +  Sensitive: + + + |
|  | Cucumber | Zhu *et al*. (2015) | 28 | 0.3 mM Na silicate | 75 mM NaCl | 15 days | 1 month | Hydroponics | Sensitive: + + +  Tolerant: + + + + | Both cultivars: + + + + |
|  | Wheat | Alzahrani *et al*. (2018) | 20 | 4 mM K silicate | 200 mM NaCl | 45 days | 60 days | Soil | + + + | + + + |
|  | Pepper | Manivannan *et al*. (2016) | 19 | 1.8 mM K silicate | 50 mM NaCl | 15 days | 3 weeks | Hydroponics | + + + | + + + + |
|  | *Lonicera japonica* | Gengmao *et al*. (2015) | 13 | 0.5 g L^-1^ K silicate | 200 mM NaCl | 30 and 40 days | 70 days after emergence | Hydroponics | + + + | N.s. |
|  | Rice | Farooq *et al*. (2015) | 12 | 150 mg kg^-1^ Na silicate | 10 dS m^-1^ | From 45 days old to booting stage | Booting stage | Soil | Tolerant: + + +  Sensitive: + + | Both cultivars: + + + + |
|  | Cucumber | Ouzounidou *et al*. (2016) | 12 | 2.25 mM K silicate (foliar spray every 10 days) | 150 mM NaCl | 2-3 months | Harvested when fruits ripe | Soil | Both cultivars: + + + | Sensitive: + + + +  Tolerant: + + + |
|  | Pea | Shahid *et al*. (2015) | 10 | 150 mg L^-1^ K silicate (foliar spray 5 and 10 days after Na stress) | 6 dS m^-1^ | 25 and 60 days | 90 days after germination | Hydroponics | + + + | + + |
|  | Chicory | D’Imperio *et al*. (2018) | 9 | 3.6 mM K meta-silicate | 50 mM NaCl | 6 weeks | 2 months | Hydroponics | N.s. | N.s. |
|  | *Brassica juncea* | Siddiqui *et al*. (2018) | 3 | 0.8 mM Na silicate (foliar spray 20 DAS) | 150 mM NaCl | 30 days | 45 days old | Soil | + + + + | + + + + |
|  | Cucumber | Zhu *et al*. (2019) | 3 | 0.3 mM Si | 75 mM Na | 10 and 15 days | 15 days after 2-leaf stage | Hydroponics | + + + | + + + |
|  | Pepper | Akhoundnejad *et al*. (2018) | 1 | 2 mM K silicate | 150 mM NaCl | 30 days | 60 days | Hydroponics | Both cultivars: + + | Tolerant: n.s.  Sensitive: + + |
|  | *Acacia gerrardii* Benth | Al-Huqail *et al*. (2019) | 1 | 2 mM K silicate | 200 mM NaCl | 8 weeks | 3 months | Hydroponics | + + + | + + + |
|  | Date palm | Jana *et al*. (2019) | 1 | 5 mM Na silicate | 300 mM NaCl | 8 weeks | 120 days old | Soil | N.s. | N.s. |
|  | Maize | Khan *et al*. (2018) | 1 | 2 mM silicic acid (5 days before Na) | 60 mM NaCl | 40 days | 50 days | Hydroponics | Tolerant: +  Sensitive: + + + | Tolerant: + + +  Sensitive: + + |
|  | Tomato | Costan *et al*. (2019) | 0 | 2 mM K silicate | 50 mM NaCl | 6 months | 6 months after 2-leaf stage | Hydroponics | N.s. | N.s. |
|  | Potato | (Kafi *et al*., 2019) | 0 | 1000 ppm SiO_2_ (foliar spray 40 and 50 days after planting) | 12 dS m^-1^ | 27 days | 57 days and 5 months | Soil | + | + + + |
| Photosynthesis | Sorghum | Liu *et al*. (2015) | 52 | 1.67 mM Na silicate (added 3 days before stress) | 100 mM NaCl | 2 hours | 12 days | Hydroponics | Unknown | + + |
|  | Okra | Abbas *et al*. (2015) | 39 | 150 mg L^-1^ silicic acid (foliar spray 1 week after stress) | 6 dS m^-1^ | 2 weeks | 6 weeks | Hydroponics | Both cultivars: + + + + | Both cultivars: + + + + |
|  | Cucumber | Wang *et al*. (2015) | 33 | 0.8 mM silicic acid 6 days before stress | 65 mM NaCl | 1.5, 3.5, 7.5 days | 17.5, 19.5, 23.5 days | Hydroponics | 7.5 d: + + | 1.5 d: n.s.  3.5 d: n.s.  7.5 d: + + + |
|  | Cucumber | Zhu *et al*. (2015) | 28 | 0.3 mM Na silicate | 75 mM NaCl | 15 days | 1 month | Hydroponics | Sensitive: + + +  Tolerant: + + + + | Both cultivars: + + + |
|  | Tomato | Li *et al*. (2015) | 25 | 2 mM Na silicate | 150 mM Na | 15 days | 15 days after 6-leaf stage | Hydroponics | + + + | + + + |
|  | Wheat | Alzahrani *et al*. (2018) | 20 | 4 mM K silicate | 200 mM NaCl | 45 days | 60 days | Soil | + + + | + + + |
|  | Pepper | Manivannan *et al*. (2016) | 19 | 1.8 mM K silicate | 50 mM NaCl | 15 days | 3 weeks | Hydroponics | + + + | + + + + |
|  | *Lonicera japonica* | Gengmao *et al*. (2015) | 13 | 0.5 g L^-1^ K silicate | 200 mM NaCl | 30 and 40 days | 70 days | Hydroponics | + + + | N.s. |
|  | Rice | Farooq *et al*. (2015) | 12 | 150 mg kg^-1^ Na silicate | 10 dS m^-1^ | From 45 days old to booting stage | Booting stage | Soil | Tolerant: + + +  Sensitive: + + | Both cultivars: + + + + + |
|  | Cucumber | Ouzounidou *et al*. (2016) | 12 | 2.25 mM K silicate (foliar spray every 10 days) | 150 mM NaCl | 2-3 months | When fruits ripe | Soil | Both cultivars: + + + | Sensitive: + + +  Tolerant: + + + + |
|  | Pea | Shahid *et al*. (2015) | 10 | 150 mg/L K silicate (foliar spray 5 and 10 days after Na stress) | 6 dS m^-1^ | 25 and 60 days | 90 days | Hydroponics | + + + | + + + |
|  | Chicory | D’Imperio *et al*. (2018) | 9 | 3.6 mM K meta-silicate | 50 mM NaCl | 6 weeks | 2 months | Hydroponics | N.s. | N.s. |
|  | Rice | Farooq *et al*. (2019) | 7 | 150 mg kg^-1^ Na silicate | 10 dS m^-1^ | Whole experiment | Booting stage | Soil | + + | + + + + + |
|  | *Phaseolus vulgaris* | Rady *et al*. (2019) | 3 | 6 mM K meta-silicate (foliar spray, 25, 35, 45 DAS) | 150 mM NaCl | 30 days | 55 days | Hydroponics | + + + | + + + |
|  | *Brassica juncea* | Siddiqui *et al*. (2018) | 3 | 0.8 mM Na silicate (foliar spray 20 DAS) | 150 mM NaCl | 30 days | 45 days | Soil | + + + + | + + + + |
|  | Cucumber | Zhu *et al*. (2019) | 3 | 0.3 mM Si | 75 mM Na | 10 and 15 days | 15 days after 2-leaf stage | Hydroponics | + + + | + + + |
|  | Pepper | Akhoundnejad *et al*. (2018) | 1 | 2 mM K silicate | 150 mM NaCl | 30 days | 60 days | Hydroponics | Both cultivars: + + | Tolerant: n.s.  Sensitive: + + |
|  | *Acacia gerrardii* Benth | Al-Huqail *et al*. (2019) | 1 | 2 mM K silicate | 200 mM NaCl | 8 weeks | 3 months | Hydroponics | + + + | + + + |
|  | Date palm | Jana *et al*. (2019) | 1 | 5 mM Na silicate | 300 mM NaCl | 8 weeks | 120 days | Soil | N.s. | N.s. |
|  | Maize | Khan *et al*. (2018) | 1 | 2 mM silicic acid (5 days before stress) | 60 mM NaCl | 40 days | 50 days | Hydroponics | Tolerant: +  Sensitive: + + + | Both cultivars: n.s. |
|  | Tomato | Costan *et al*. (2019) | 0 | 2 mM K silicate | 50 mM NaCl | 6 months | 6 months after 2-leaf stage | Hydroponics | N.s. | N.s. |
|  | Potato | (Kafi *et al*., 2019) | 0 | 1000 ppm SiO_2_ (foliar spray 40 and 50 days after planting) | 12 dS m^-1^ | 27 days | 57 days and 5 months | Soil | + | + + + + + |
| Chlorophyll content | Sorghum | Yin *et al*. (2016) | 42 | 0.83 mM silicic acid | 100 mM NaCl | 1, 3, and 7 days | 16, 30, and 24 days | Hydroponics | 1 d and 3 d: n.s.  7 d: + + + + | 1 d: n.s.  3 d: + + + +  7 d: + + + |
|  | Okra | Abbas *et al*. (2015) | 39 | 150 mg L^-1^ silicic acid (foliar spray 1 week after stress) | Electrical conductivity of 6 dS m-1 | 1 month after sowing for 2 weeks | 6 weeks | Hydroponics | Both cultivars: + + + + | Tolerant: + + + + +  Sensitive: + + + |
|  | Chickpea | Garg and Bhandari, (2016) | 38 | 4 mM K silicate | 100 mM NaCl | 9 weeks | 80 days | Soil | Both cultivars: + + | Tolerant cultivar: +  Sensitive cultivar: + + |
|  | Cucumber | Wang *et al*. (2015) | 33 | 0.8 mM silicic acid 6 days before stress | 65 mM NaCl | 1.5, 3.5, 7.5 days | 17.5, 19.5, 23.5 days | Hydroponics | + + after 7.5 days stress | 1.5 d: n.s.  3.5 d: + + + + +  7.5 d: + + + |
|  | Tomato | Li *et al*. (2015) | 25 | 2 mM Na silicate | 150 mM Na | 15 days | 15 days after 6-leaf stage | Hydroponics | + + + | + + + |
|  | Carnation | Soundararajan *et al*. (2015) | 18 | 50 mg K silicate | 100 mM NaCl | 30 days | 30 days | Agar | + + + + | + + + |
|  | Rice | Farooq *et al*. (2015) | 12 | 150 mg kg^-1^ Na silicate | 10 dS m^-1^ | From 45 days old to booting stage | Booting stage | Soil | Tolerant: + + +  Sensitive: + + | Both cultivars: + + + + + |
|  | Rice | Mahdieh *et al*. (2015) | 12 | 3 mM Na silicate | 100 mM NaCl | 4 days | 30 days old | Hydroponics | Both cultivars: n.s. | Sensitive: n.s.  Tolerant: – – – – – |
|  | Cucumber | Ouzounidou *et al*. (2016) | 12 | 2.25 mM K silicate (foliar spray every 10 days) | 150 mM NaCl | 2-3 months | Harvested when fruits ripe | Soil | Both cultivars: + + + | Both cultivars: + + + |
|  | Pea | Shahid *et al*. (2015) | 10 | 150 mg L^-1^ K silicate (foliar spray 5 and 10 days after stress) | 6 dS m^-1^ | 25 days | 90 days | Hydroponics | + + + | + + + + |
|  | Cucumber | Yin *et al*. (2019) | 10 | 0.3 mM Na meta-silicate | 75 mM NaCl | 5, 10, 15 days | 12, 17, 22 days after 2-leaf stage | Hydroponics | 15 d: + + + | 15 d: + + + |
|  | Gooseberry | Rezende *et al*. (2017) | 7 | 1 g L^-1^ silicic acid | 1 % NaCl | 30 days | Unknown (explants) | Phytagel | Unknown | (Positive effect of Si only seen at lower Na level)  – – – – – |
|  | Maize | Bosnic *et al*. (2018) | 3 | 1.5 mM silicic acid (7 days before stress) | 40 mM NaCl | 3 weeks | 5 weeks | Hydroponics | + + + | + + + |
|  | *Phaseolus vulgaris* | Rady *et al*. (2019) | 3 | 6 mM K meta-silicate (foliar spray, 25, 35, 45 DAS) | 150 mM NaCl | 30 days | 55 days | Hydroponics | + + + | + + + |
|  | *Brassica juncea* | Siddiqui *et al*. (2018) | 3 | 0.8 mM Na silicate (foliar spray 20 DAS) | 150 mM NaCl | 30 days | 45 days | Soil | + + + + | + + + + + |
|  | Wheat | Sienkiewicz-Cholewa *et al*. (2018) | 3 | Exp 1: 1 mM silicic acid  Exp 2: 1.5 mM | Exp 1: 70 mM NaCl  Exp 2: 100 mM | 10 d | 12 days | Hydroponics | Exp 1: n.s.  Exp 2: + | Exp 1: + +  Exp 2: + + + |
|  | Wheat | Daoud *et al*. (2018) | 2 | 0.78 mM Na meta-silicate | 120 mM NaCl | 10, 21, 68, 108 days | 22, 43, 80 and 120 days | Hydroponics | All growth stages: + + + | All growth stages: + + + |
|  | Coriander | Al-Garni *et al*. (2019) | 1 | 150 mL 0.1% w/v K silicate (foliar spray 1 week after stress) | 75 mM NaCl | 3 weeks | 5 weeks | Soil | + + + | + + + + + |
|  | *Acacia gerrardii* Benth | Al-Huqail *et al*. (2019) | 1 | 2 mM K silicate | 200 mM NaCl | 8 weeks | 3 months | Hydroponics | + + + | + + + |
|  | Maize | Khan *et al*. (2018) | 1 | 2 mM silicic acid (5 days before stress) | 60 mM NaCl | 40 days | 50 days | Hydroponics | Tolerant: +  Sensitive: + + + | Both cultivars: + + + + |
|  | Maize | Delavar *et al*. (2018) | 0 | 2 mM Na meta-silicate (2 weeks before stress) | 150 mM NaCl | 2 weeks | 5 weeks | Hydroponics | + + + | – – |
|  | Canola | El-Shazoly (2019) | 0 | 1 mM Na silicate | 90 mM NaCl | 8 days | 8 days | Petri dish | + + + + + | + + + |
|  | Potato | (Kafi *et al*., 2019) | 0 | 1000 ppm SiO_2_ (foliar spray 40 and 50 days after planting) | 12 dS m^-1^ | 27 days | 57 days and 5 months | Soil | + | + + + + + |
|  | Rice | Lekklar *et al*. (2019) | 0 | 0.5 mM silicic acid | 60 mM NaCl | 7 and 14 days | 4 and 5 weeks | Hydroponics | 7 d: + + + +  14 d: + + + | 7 d: + + + +  14 d: + + + + |
|  | Wheat | Mushtaq *et al*. (2019) | 0 | 0.1 g L^-1^ Na silicate or silicic acid | 100 mM NaCl | 30 days | 30 days after 3^rd^ leaf stage | Hydroponics | All cultivars and Si fertilisers: n.s. | All cultivars and Si fertilisers: + + |
| Carotenoid content | Okra | Abbas *et al*. (2015) | 39 | 150 mg L^-1^ silicic acid (foliar spray 1 week after stress) | 6 dS m^-1^ | 2 weeks | 6 weeks | Hydroponics | Both cultivars: + + + + | Tolerant: + + + +  Sensitive: + + + |
|  | Tomato | Li *et al*. (2015) | 25 | 2 mM Na silicate | 150 mM Na | 15 days | 15 days after 6-leaf stage | Hydroponics | + + + | + + + |
|  | Gooseberry | Rezende *et al*. (2017) | 7 | 1 g L^-1^ silicic acid | 1 % NaCl | 30 days | Unknown (explants) | Phytagel | Unknown | (Positive effect of Si only seen at lower Na level)  – – – – – |
|  | *Phaseolus vulgaris* | Rady *et al*. (2019) | 3 | 6 mM K meta-silicate (foliar spray, 25, 35, 45 DAS) | 150 mM NaCl | 30 days | 55 days | Hydroponics | + + + | + + + |
|  | Wheat | Sienkiewicz-Cholewa *et al*. (2018) | 3 | Exp 1: 1 mM silicic acid  Exp 2: 1.5 mM | Exp 1: 70 mM NaCl  Exp 2: 100 mM | 10 d | 12 d | Hydroponics | Exp 1: n.s.  Exp 2: + | Both experiments: n.s. |
|  | Wheat | Daoud *et al*. (2018) | 2 | 0.78 mM Na meta-silicate | 120 mM NaCl | 10, 21, 68, 108 days | 22, 43, 80 and 120 days | Hydroponics | All growth stages: + + + | All growth stages: + + + + |
|  | Coriander | Al-Garni *et al*. (2019) | 1 | 150 mL 0.1% w/v K silicate (foliar spray 1 week after stress) | 75 mM NaCl | 3 weeks | 5 weeks | Soil | + + + | + + + + + |
|  | *Acacia gerrardii* Benth | Al-Huqail *et al*. (2019) | 1 | 2 mM K silicate | 200 mM NaCl | 8 weeks | 3 months | Hydroponics | + + + | + + + |
|  | Maize | Delavar *et al*. (2018) | 0 | 2 mM Na meta-silicate (2 weeks before stress) | 150 mM NaCl | 2 weeks | 5 weeks | Hydroponics | + + + | + + + + + |
|  | Canola | El-Shazoly (2019) | 0 | 1 mM Na silicate | 90 mM NaCl | 8 days | 8 days | Petri dish | + + + + + | + + + |
|  | Potato | (Kafi *et al*., 2019) | 0 | 1000 ppm SiO_2_ (foliar spray 40 and 50 days after planting) | 12 dS m^-1^ | 27 days | 57 days and 5 months | Soil | + | + + + + + |
|  | Rice | Lekklar *et al*. (2019) | 0 | 0.5 mM silicic acid | 60 mM NaCl | 7 and 14 days | 4 and 5 weeks | Hydroponics | 7 d: + + + +  14 d: + + + | 7 d: + + + +  14 d: + + + + |

*Search criteria:

- Search was carried out using Web of Science ([www.webofknowledge.com](http://www.webofknowledge.com)) database between 17/12/19 and 23/12/19 using the following search terms: Silicon AND drought; Silicon AND “osmotic stress”; Silicon AND salinity; Silicon AND “salt stress”, covering the last 5 years (01/01/2015 to 31/12/2019).
- Citation scores are based on numbers retrieved from web of science on 10/01/20
- Research articles only were included
- All papers from 2019 and 2018 were included, earlier publications were only included if cited >10-fold

**Effect size guide:

- Very large: under stress, levels with Si surpass those for control conditions **(+++++/-----)** with ‘+’ and ‘-‘ denoting positive and negative change respectively
- Large: under stress, levels with Si are similar to those for control conditions **(++++/----)**
- Medium: under stress, levels with Si are ~50% of those for control conditions **(+++/ ---)**
- Small: under stress, levels with Si are ~25% of those for control conditions **(++/--)**
- Very small: under stress, levels with Si are less than 25% of those for control conditions **(+/-)**
- No significant effect **(n.s.)**
